# Engineering Supramolecular Systems with a Bis(pyridyl)azine Derivative and Different Hydrogen and Halogen Donors

**DOI:** 10.1021/acs.cgd.5c01135

**Published:** 2025-12-26

**Authors:** Mayra S. Coutinho, Thomaz de A. Costa, Alan Imperatori, Andrei A. Patrascu, Isabela Man, Maria G. F. Vaz, Simona Nica, Marius Andruh, Pedro N. Batalha

**Affiliations:** 1 Chemistry Department, Instituto Federal do Paraná, Palmas, Paraná 85690-740,Brazil; 2 Instituto de Química, 426050 Universidade Federal Fluminense, Centro, Niterói, Rio de Janeiro 24210-201,Brazil; 3 Inorganic Chemistry Laboratory, Faculty of Chemistry, 61783 University of Bucharest, Bd. Regina Elisabeta 4-12, Bucharest 030018, Romania; 4 “C. D. Nenitzescu” Institute of Organic and Supramolecular Chemistry,Romanian Academy, Splaiul Independentei 202B, Bucuarest 060023, Romania

## Abstract

Cocrystal engineering has become an essential strategy in materials science, enabling the design of new solid-state systems through the rational combination of organic components guided by noncovalent interactions. In this study, the cocrystallization of a bis-pyridyl-azine substrate with three different coformers, namely, 1,3,5-triiodo-2,4,6-trifluorobenzene, 1,2-diiodotetrafluorobenzene, and 4,4′-biphenol, led to the formation of five new cocrystal systems. The supramolecular architectures of these materials were investigated with a focus on the role of halogen bonding and hydrogen bonding in crystal packing. All observed noncovalent interactions were investigated using density functional theory (DFT) calculations carried out with the ωB97XD functional and the def2-TZVPP. The results emphasize the influence of molecular recognition in the self-assembly process and reveal how halogen and hydrogen bonds act as key driving forces in the formation and stabilization of these cocrystals.

## Graphical abstract

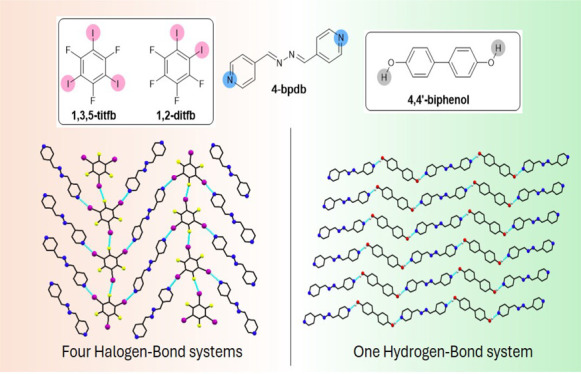

## Introduction

Cocrystal engineering is a powerful strategy in materials science, since the cocrystallization of two or more organic species into a single crystalline phase is a strategy widely valued for the possibility to generate new systems with enhanced physical properties beyond those of their individual components.^1,2^ Their exceptional characteristics, including ease preparation, tunable structures, large-area solution processing, excellent flexibility, and lightweight nature, make them useful in engineering, chemical processes, and materials design.^2–4^ The assembly of cocrystals is strongly influenced by molecular recognition and supramolecular self-assembly driven by noncovalent interactions. These interactions include charge transfer, hydrogen bonding (H-bonding), halogen bonding (X-bonding), π–π stacking, and van der Waals forces, which play a crucial role in the cocrystallization process.^5–7^ This approach has gained prominence in chemistry and materials science due to its potential to enhance physicochemical properties, such as luminescence,^8^ metallic conductivity,^9^ ferroelectricity,^10^ nonlinear optics,^11^ and photophysical interaction with the matter.^12^

In recent decades, X-bonding has emerged as a powerful tool in crystal engineering and supramolecular chemistry, extending beyond the well-established H-bonding.^13,14^ This noncovalent interaction occurs between an electrophilic region on a halogen atom, known as the σ-hole, and a nucleophilic site on another atom, resulting in the formation of a halogen bond.^15–17^ When the σ-hole interacts with a lone pair on a heteroatom, such as nitrogen, the interaction is classified as *n*-type.^18^ The electronegativity of the halogen atom influences the strength of the halogen bonding. The presence of electron-withdrawing substituents, such as fluorine, cyano, or nitro groups in the halogen donor substrate, can further enhance it.^19,20^ X-bonding has been acknowledged as a competitive force alongside H-bonding in guiding the assembly of multicomponent systems into supramolecular architectures.^21,22^ Recent researches have demonstrated that cocrystals can integrate both H-bonding and X-bonding interactions within a single supramolecular aggregate.^23,24^ The strongest intermolecular interactions are represented by conventional hydrogen bonds (A–H···B), where A and B are elements such as N, O, or F.^25^ In contrast, nonconventional hydrogen bonds, such as C–H···O/N, are significantly weaker than their conventional counterparts.^26,27^ X-bonding and H-bonding both form structures composed of subunits known as supramolecular synthons, which may be classified as either homosynthons (formed by self-assembling identical units) or heterosynthons (containing different acceptor–donor molecules).^28,29^ However, despite its potential, X-bonding has not gained the same level of prominence as H-bonding. This is partly due to its dependence on specific halogen atoms (F, Cl, Br, and I) in the coformer(s), which imposes structural constraints.^30^ The broader applicability and versatility of H-bonding have made it the preferred interaction in crystal engineering and supramolecular design.^31^ On the other hand, in some instances, X-bonding cocrystals have been shown to exhibit greater stability than H-bonding cocrystals, which have attracted more attention for this particular type of system.^32,33^

When considering a standard system to serve as an optimal acceptor, substrates containing the pyridine moiety stand out as some of the most extensively studied and reliable acceptors for both halogen and hydrogen bonds, distinguished by their exceptional performance.^34–36^ These systems have become essential benchmarks for evaluating halogen and hydrogen-bond donors, as well as for assessing the competitiveness of various acceptor species.^23,37^ The bis-pyridyl-azine ligand 1,4-bis­(4-pyridyl)-2,3-diaza-1,3-butadiene (**4-bpdb**) is widely recognized for its conformational flexibility, making it a valuable supramolecular building block in materials chemistry and crystal engineering.^38^ Its structural adaptability has facilitated its extensive application in the design of coordination architectures, including the synthesis of coordination polymers (CPs).^39^ However, despite its favorable properties, its use in combination with perfluorinated iodobenzenes remains limited,^40^ and its incorporation with bisphenols is rarely explored. This underutilization presents an opportunity for further investigation into its potential interactions and applications in supramolecular assembly.

Recently, some of us investigated halogen bonding interactions using 1,2-diiodotetrafluorobenzene (**1,2-ditfb**), 1,4-diiodotetrafluorobenzene (**1,4-ditfb**), and 1,3,5-triiodo-2,4,6-trifluorobenzene (**1,3,5-titfb**) as donor groups, with 1,3-bis­(4-pyridyl)­azulene incorporated as an acceptor component. This study led to the formation of three novel cocrystals featuring I···N heterosynthons.^41^ Systems incorporating hydrogen-bond donors, such as biphenol, with 1,3-bis­(4-pyridyl)­azulene as the acceptor were also explored.^42^ In these cases, both conventional (O–H···N) and nonconventional (C–H···N) hydrogen bonding interactions were observed.

In this work, we describe our results exploring OH···N and I···N interactions by using a *bis*-pyridyl-azine derivative (**4-bpdb**) as a building block for the construction of new cocrystalline systems (Scheme 1). Weak interactions, such as C–H···N, C–H···F, and π-π stacking, have also been shown to be essential for these systems, as discussed below.

**Scheme 1:**
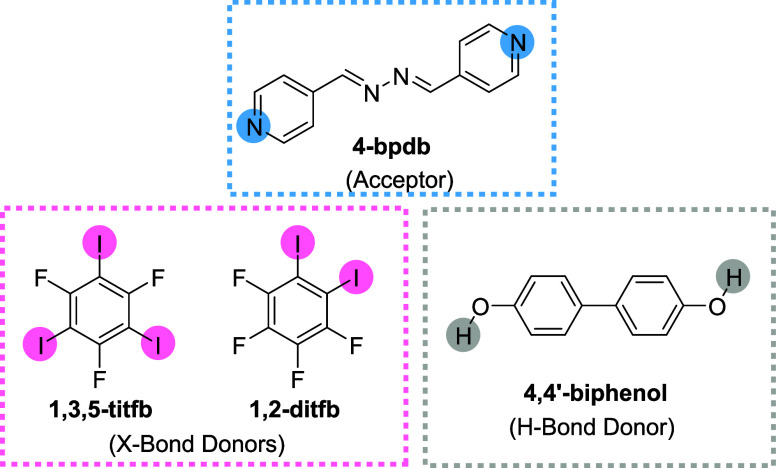
Chemical Structures of the Halogen and Hydrogen-Bond Donors and Acceptor Used in This Study

## Experimental Section

### Materials and Instrumentation

Commercially available chemicals were used without further purification. 1,4-*bis*(4-pyridyl)-2,3-diaza-1,3-butadiene (**4-bpdb**) was synthesized following an adaptation of the procedure reported by Kurisingal et al.^43^ as described below.

X-ray diffraction measurements were performed on a Rigaku XtaLAB Synergy-S diffractometer operating with Mo–Kα (λ = 0.71073 Å) microfocus sealed X-ray tube. The structures were solved by direct methods and refined by full matrix least-squares procedures on *F* ^2^ ^44^ using SHELX-2018 crystallographic software package.^45,46^ All of the non-H atoms of the donor molecules were refined anisotropically. The powder X-ray diffraction data were measured on a Proto AXRD benchtop using Cu Kα radiation with a wavelength of 1.54059 Å in the range 5–30° (2θ). IR spectra (KBr pellets) were recorded on a Tensor 37 spectrophotometer in the 4000–400 cm^–1^ region. Elemental analysis was not performed, as the identity, stoichiometry, and structural integrity of all cocrystals were unambiguously confirmed by single-crystal X-ray diffraction. Crystallographic data for the structures are deposited in the Cambridge Crystallographic Data Centre, deposition numbers CCDC 2477261–2477265. Complementary data can be found in the Supporting Information file.

### Synthesis of 1,4-Bis­(4-pyridyl)-2,3-diaza-1,3-butadiene (**4-bpdb**)

Pyridine-4-carboxaldehyde (1 mmol, 94 μL) was added dropwise under stirring into a flask containing hydrazine monohydrochloride (0.5 mmol, 34 mg) in 3 mL of water. The solution was stirred at room temperature for 5 min. The yellow precipitate thus obtained was collected, washed with cold ethanol, and dried in the air. Yield: 90%. Mp: 182 °C. Selected IR ν (cm^–1^; ATR): 3030 and 2949 (Csp^2^-H, axial); 1629 (C=N, asymmetric axial); 1595 (C=N, symmetric axial); 1083 (N–N, axial). ^1^H NMR (500 MHz, DMSO-*d*_6_): δ 8.74 (dd, 4H), 8.66 (s, 2H), 7.80 (dd, 4H) (Figures S1 and S2).

### Cocrystal Synthesis

All systems were prepared by using the same **4-bpdb** substrate. Cocrystals **1**, **2**, and **3** were synthesized with the same halogen-bond donor, 1,3,5-triiodo-2,4,6-trifluorobenzene (**1,3,5-titfb**). The main differences among these syntheses arise from the distinct molar ratios and solvent systems employed. Cocrystals **4** and **5** were obtained by mixing **4-bpdb** with 1,2-diiodotetrafluorobenzene (**1,2-ditfb**) and **4,4′-biphenol**, as halogen- and hydrogen-bond donors, respectively. Table 1 summarizes the molar ratios and solvent systems used in the preparation of compounds **1–5.**

**Table 1:** Molar Equivalent Ratios and Solvent Systems Used for Compounds **1–5**

cocrystal	acceptor (A)	donor (D)	equivalents A:D	solvent
**1**	**4-bpdp**	**1,3,5-titfb**	3:2	MeOH
**2**	**4-bpdp**	**1,3,5-titfb**	3:2	MeOH/CHCl_3_
**3**	**4-bpdp**	**1,3,5-titfb**	1:1	MeOH/CHCl_3_
**4**	**4-bpdp**	**1,2-ditfb**	1:1	MeOH/CHCl_3_
**5**	**4-bpdp**	**4,4′-biphenol**	1:1	MeOH/CHCl_3_

#### Triiodotrifluorobenzene Cocrystal, (**4-bpdb**)-(**1,3,5-titfb**), **1**

In two different flasks, 1,4-bis­(4-pyridyl)-2,3-diaza-1,3-butadiene (6.3 mg, 0.03 mmol) and 1,3,5-triiotrifluorobenzene (10.2 mg, 0.02 mmol) were separately dissolved in 10.0 and 5.0 mL of CH_3_OH, respectively. The 1,3,5-triiotrifluorobenzene solution was then added to the 1,4-bis­(4-pyridyl)-2,3-diaza-1,3-butadiene one in a dropwise manner. The resulting mixture was stirred at room temperature for 2 h and left to crystallize. Orange crystals were obtained by slow evaporation after 3 days at room temperature. Selected IR ν (cm^–1^; KBr): 3038­(vw), 2938­(vw), 1684­(w), 1627­(m), 1593(s), 1562(s), 1394­(vs), 1304­(m), 1041(s), 950­(m), 812(s), 678­(m), 651­(m), 509(s).

#### Triiodotrifluorobenzene Cocrystal, (**4-bpdb**)-(**1,3,5-titfb**), **2**

To a solution of 1,4-bis­(4-pyridyl)-2,3-diaza-1,3-butadiene (6.3 mg, 0.03 mmol) in CH_3_OH (2 mL) was added 1,3,5-triiotrifluorobenzene (10.2 mg, 0.02 mmol) dissolved in CHCl_3_ (2 mL). The resulting mixture was stirred at room temperature for 2 h. Yellow crystals were obtained by slow evaporation after 1 day at room temperature. Selected IR ν (cm^–1^; KBr): 3040­(vw), 2947­(vw), 1685­(w), 1629­(m), 1595(s), 1560(s), 1395­(vs), 1306­(m), 1037(s), 950­(m), 810(s), 678­(m), 648­(m), 508­(m).

#### Triiodotrifluorobenzene Cocrystal, (**4-bpdb**)-(**1,3,5-titfb**), **1** and **3**

To a solution of 1,4-bis­(4-pyridyl)-2,3-diaza-1,3-butadiene (6.3 mg, 0.03 mmol) in CH_3_OH (2 mL) was added 1,3,5-triiotrifluorobenzene (15.3 mg, 0.03 mmol) dissolved in CHCl_3_ (2 mL). The resulting mixture was stirred at room temperature for 2 h. Orange crystals were obtained by slow evaporation after 1 day at room temperature. Selected IR ν (cm^–1^; KBr): 3038­(vw), 2939­(m), 1685­(w), 1628­(m), 1593(s), 1562(s), 1393­(vs), 1304­(m), 1041(s), 950­(m), 812(s), 677­(m), 651­(m), 509­(m).

#### 1,2-Diiototetrafluorobenzene Cocrystal, (**4-bpdb**)-(**1,2-ditfb**), **4**

To a solution of 1,4-bis­(4-pyridyl)-2,3-diaza-1,3-butadiene (10.5 mg, 0.05 mmol) in CH_3_OH (2 mL) was added 1,2-diiototetrafluorobenzene (20.1 mg, 0.05 mmol) dissolved in CHCl_3_ (2 mL). The resulting mixture was stirred at room temperature for 2 h. Orange crystals were obtained by slow evaporation after 1 day at room temperature. Selected IR ν (cm^–1^; KBr): 3033­(vw), 2962­(vw), 1686­(w), 1628­(m), 1594(s), 1549­(m), 1482(s), 1431­(vs), 1409(s), 1306­(m), 1009­(m), 967­(m), 810­(vs), 761­(w), 677­(m), 510­(m).

#### 4,4′-Biphenol Cocrystal, (**4-bpdb**)-(**4,4′-biphenol**), **5**

To a solution of 1,4-bis­(4-pyridyl)-2,3-diaza-1,3-butadiene (10.5 mg, 0.05 mmol) in CH_3_OH (4 mL) was added **4,4′-biphenol** (9.3 mg, 0.05 mmol) dissolved in CHCl_3_ (2 mL). The resulting mixture was stirred at room temperature for 2 h. Yellow crystals were obtained by slow evaporation after 1 day at room temperature. Selected IR ν (cm^–1^; KBr): 3051­(w), 3001­(w), 2947­(w), 2789­(w), 2722­(w), 2654­(w), 1696­(w), 1601(s), 1579(s), 1554(s), 1495(s), 1414­(vs), 1300(s), 1256(s), 1223(s), 957­(m), 811(s), 779(s), 726­(m), 681­(m), 511­(m).

### Computational Details

Calculations were performed on donor/acceptor dimer types that were extracted from the X-ray measurement diffraction data, along the desired interactions, mainly halogen ones (I···I, I···F, I···N). No further optimizations were performed, with the atoms that have the atomic positions as resulted from the diffraction data. The density theory calculations (DFT) were performed with the ω97-XD functional^47^ combined with def2-TZVPP basis set for all atoms and effective core potentials applied to iodine atom as implemented in the Gaussian16 software.^48^ This combination of functional-basis set was shown to give low errors for similar systems (e.g., 1,3,5-triiotrifluorobenzene and ammonia).^49^ All calculations were done in vacuum. The electrostatic potential surface (ESP) maps (iso density = 0.001 au) were constructed. The interaction or binding energy in the donor–acceptor complexes was computed as the difference in energy between the dimer complex in a donor/acceptor relationship (e.g., halogen–XBDA, hydrogen–HBDA) and the sum of the energies of the individual components. The value was corrected for basis set superposition error (BSSE) by the counterpoise technique:^50^

$$\Delta E_{\mathrm{int}}=E_{\mathrm{BDA}}-(E_{\mathrm{BD}}+E_{\mathrm{BA}})$$

where *E*_BDA_ is the energy of the bond donor–acceptor complex (BDA), *E*_BD_ is the energy of the bond donor, and *E*_BA_ is the energy of the bond acceptor. In the trimer complexes, the same relation was used but the donor or acceptor is the complex previously calculated. Atoms in molecule (AIM) bond paths and their associated critical points were located and their densities evaluated with the aid of the multiwfn program.^51^

The natural bond orbital (NBO) analysis is used to give insights into the nature of interactions existing between the donor orbital and acceptor orbital, which is expressed in terms of second-order perturbation energy *E* ^(2)^ or stabilization energy. It was done at the same level of theory. NBO transforms the canonical delocalized molecular orbitals from DFT calculations into localized orbitals. The mixing of donors and acceptors leads to an overall energy lowering (“stabilization”). The delocalizing/stabilizing interaction can be treated, as already mentioned, via the second-order perturbation energy approach: *E* ^(2)^ = *n*_i_|*F*_ij_|^2^/Δ*E*, where *n*_i_ is the population of a donor orbitals, *F*_*ij*_ is the Fock matrix element for the interacting orbitals *i* and *j*, and Δ*E* is the energy gap between these orbitals. The higher the stabilization energy, the stronger the interaction between the donor orbital and the acceptor orbital.

## Results and Discussion

### Synthesis and Crystallization

The *bis*-pyridyl-azine (**4-bpdb**) precursor was obtained in high yield (90%) following a modified synthetic procedure, using water as the solvent,^43^ and its crystallographic structure has been reported.^52^ The molecular structures of the acceptor (**4-bpdb**) and the halogen (XB) and hydrogen (HB) bonding donors are presented in Scheme 1. The cocrystals were prepared at room temperature by mixing their respective components and allowing the solution to stand for slow evaporation. Suitable crystals for X-ray determination were obtained within 1 to 3 days.

First, **4-bpdb** was cocrystallized with **1,3,5-titfb** under three different conditions, yielding the cocrystal **(4-bpdb)·(1,3,5-titfb)** as three polymorphs, labeled **1**, **2**, and **3**. The experiments were conducted using the following component ratios: 3:2 of **4-bpdb**/**1,3,5-titfb** in methanol, 3:2 of **4-bpdb/1,3,5-titfb** in a methanol/chloroform mixture, and 1:1 of **4-bpdb**/**1,3,5-titfb** in a methanol/chloroform mixture. In the experiments using a 3:2 ratio of **4-bpdb**/**1,3,5-titfb**, polymorphs **1** and **2** were isolated. In contrast, a stoichiometric ratio between **4-bpdb** and **1,3,5-titfb** in a methanol/chloroform mixture yielded **1**, together with polymorph **3.**

Furthermore, cocrystallization was carried out using the **1,2-ditfb** XB donor in a 1:1 ratio with a methanol/chloroform mixture, resulting in the crystallization of the new system **4,** (**4-bpdb**)·(**1,2-ditfb**)_2_. In general, XB-type interactions were observed for both XB donors when **4-bpdb** was used as the XB acceptor. Additionally, weak C–H···N interactions were also identified in crystals **1** and **2.**

HB interactions were also investigated using **4,4′-biphenol** as the HB donor component. The cocrystallization was performed using a 1:1 ratio of **4-bpdb** to **4,4′-biphenol** in a methanol/chloroform mixture, forming the new cocrystal **5** through typical O–H···N hydrogen bonding interactions. Crystallographic data for cocrystals **1**–**3** are summarized in Table 2, while the data for cocrystals **4** and **5** are presented in Table 3.

**Table 2:** Crystal Data and Structure Refinement for Cocrystals **1**–**3**

cocrystal	1	2	3
chemical formula	C_36_H_20_F_6_I_6_N_8_	C_18_H_10_F_3_I_3_N_4_	C_18_H_10_F_3_I_3_N_4_
M (g mol^–1^)	1440.00	720.00	720.00
temperature, (K)	293(2)	293(2)	293(2)
wavelength, (Å)	0.71073	0.71073	0.71073
crystal system	*monoclinic*	*monoclinic*	*monoclinic*
space group	*P2*_*1*_ */n*	*P2*_*1*_ */n*	*P2*_*1*_ */m*
*a* (Å)	4.3210(2)	11.1536(5)	4.8433(4)
*b* (Å)	32.7785(17)	9.1596(4)	33.096(3)
*c* (Å)	30.1886(13)	21.1084(10)	6.7783(6)
α (°)	90	90	90
β (°)	92.511(4)	92.456(4)	101.230(8)
γ (°)	90	90	90
V (Å^3^)	4271.7(3)	2154.51(17)	1065.72(17)
Z	4	4	2
*D*_*c*_ (g cm^–3^)	2.239	2.220	2.244
μ (mm^–1^)	4.427	4.388	4.436
*F* (000)	2656	1328	664
θ range (deg.)	1.982–25.000	2.106–29.401	2.462–25.000
index range	–5 ≤ *h* ≤ 4	–13 ≤ *h* ≤ 13	–5 ≤ *h* ≤ 4
	–38 ≤ *k* ≤ 38	–10 ≤ *k* ≤ 10	–39 ≤ *k* ≤ 38
	–31 ≤ *l* ≤ 35	–25 ≤ *l* ≤ 25	–8 ≤ *l* ≤ 8
data collected/independent reflections	19300/7453 [R_int_ = 0.0344]	12044/3786 [R_int_ = 0.0395]	5501/1900 [R_int_ = 0.0309]
data/restraints/parameters	7453/0/505	3786/0/254	1900/0/134
GOF	1.028	1.062	1.075
final *R*_1_, w*R*_2_ [I > 2σ(I)]	0.0331, 0.0701	0.0340, 0.0820	0.0576, 0.1474
*R*_1_, w*R*_2_ (all data)	0.0488, 0.0756	0.0413, 0.0853	0.0684, 0.1546
Δρ_min_/Δρ_max_ (e Å^–3^)	0.507, −0.731	0.768, −0.802	1.374, −1.311

**Table 3:** Crystal Data and Structure Refinement for Cocrystals **4** and **5**

cocrystal	4	5
chemical formula	C_24_H_10_F_8_I_4_N_4_	C_24_H_20_N_4_O_2_
M (g mol^–1^)	1013.96	396.44
temperature, (K)	293(2)	293(2)
wavelength, (Å)	0.71073	0.71073
crystal system	*monoclinic*	*monoclinic*
space group	*P2*_*1*_ */c*	*P2*_*1*_ */c*
*a* (Å)	13.3749(5)	5.9377(4)
*b* (Å)	4.28920(10)	23.5324(13)
*c* (Å)	24.7279(9)	7.2880(5)
α (°)	90	90
β (°)	97.839(4)	95.089(6)
γ (°)	90	90
V (Å^3^)	1405.32(8)	1014.33(11)
Z	2	2
*D*_*c*_ (g cm^–3^)	2.396	1.298
μ (mm^–1^)	4.510	0.085
*F* (000)	932	416
θ range (deg.)	2.105–24.998	2.937–29.999
Index range	–15 ≤ *h* ≤ 14	–7 ≤ *h* ≤ 5
	–4 ≤ *k* ≤ 5	–27 ≤ *k* ≤ 26
	–29 ≤ *l* ≤ 29	–8 ≤ *l* ≤ 8
data collected/independent reflections	9406/2456 [R_int_ = 0.0297]	5292/1782 [R_int_ = 0.0198]
data/restraints/parameters	2456/0/181	1782/0/138
GOF	1.080	1.044
final *R*_1_, w*R*_2_ [I > 2σ(I)]	0.0214, 0.0485	0.0360, 0.0967
*R*_1_, w*R*_2_ (all data)	0.0259, 0.0509	0.0439, 0.1015
Δρ_min_/Δρ_max_ (e Å^–3^)	0.320, −0.581	0.150, −0.129

### Description of the Crystal Structures

#### Halogen-Bonded Assemblies with **1,3,5-titfb**

The cocrystallization of **4-bpdb** with the **1,3,5-titfb**, performed in a 3:2 ratio in methanol yielded yellow cocrystal **1**, upon slow evaporation at room temperature. It crystallizes in the monoclinic *P2*_1_ */n* space group containing a stoichiometric XB donor and acceptor connected through C–I···N halogen bonds. The C–I···N interaction angles ranged from 162.4 to 196.2°, as shown in Table 4. The asymmetric unit comprises four crystallographically independent molecules consisting of two **1,3,5-titfb** and two **4-bpdb** molecules. These components predominantly assemble through linear I···N halogen bonds, leading to *zigzag* chains and layers with the same architecture formed along the crystallographic *a* axis (Figure 1 and Figure S3). The **1,3,5-titfb** molecule acts as a ditopic halogen bond donor with C–I···N interaction distances ranging from 2.891 to 3.059 Å. These halogen bonds can be considered relatively strong due to the significant shortening of the interaction distance, which is approximately 13.3–18.1% shorter than the sum of the van der Waals radii of the nitrogen and iodine atoms.^53^ In addition to the C–I···N halogen bond, the presence of a C–I···F halogen bond was also observed, with C17–I2···F4 and C17–I6···F3 angles of 170.2° and 167.5°, respectively (Figure 1 and Table 4). The halogen bonded chains are connected into pairs through C–I···F contacts (C17–I2···F4 of 3.030 Å and C17–I6···F3 of 3.058 Å). Furthermore, an additional C–H···F hydrogen bond was identified, with C20–H20···F5 measuring 2.53 Å and an angle of 135° (see Table 5). The interplay of halogen–halogen interactions and C–H···F hydrogen bonds results in the formation of two-dimensional supramolecular sheets. These chains are further connected into layers with weak I···π­(C=C) and F···π­(C=C) halogen bonds resulting in an extended layered architecture (see Supporting Information, Figure S4 and Table S1). Between the layers, a weak interaction between C–H···H–C (2.36 Å) is also observed (Figure S4).

**Table 4:** Halogen Bonds Parameters for 1–4

cocrystal	D···A	d(DA)/Å	∠(C–D···A)/°	∠(D···A–C)/°	symmetry code
**1**	I1···N4	2.891	166.07	137.10	–x,1–y,1–z
	I3···N5	2.958	163.92	103.80	5/2–x,1/2+y,3/2–z
	I4···N8	2.900	196.19	135.30	
	I5···N1	3.059	162.40	100.50	x,–1+y,z
	I2···F4	3.030	170.20	159.82	–x,1–y,1–z
	I6···F3	3.058	167.50	163.57	5/2–x,–1/2+y,3/2-z
**2**	I1···N1	2.831	176.89	122.40	–3/2+x,3/2–y,1/2+z
	I2···N4	2.904	173.88	117.70	3/2+x,3/2–y,–1/2+z
**3**	I1···N1	2.902	167.00	130.20	–1–x,–y,1–z
	I3···N3	2.994	167.10	109.60	1+x,1+y,z
	I2···F3	2.932	178.9	178.10	–1+x,y,z
**4**	I1···N1	2.866	175.13	123.80	–x,2–y,2–z

**Table 5:** Selected Hydrogen Bonding Metrics for Cocrystals **1**–**3** and **5**

cocrystal	D–H···A	d(DH)/Å	H···A/Å	D···A/Å	(D–H···A)/°	symmetry code
**1**	C20–H20···F5	0.93	2.53	3.255	135	5/2–x,1/2+y,3/2–z
**2**	C10–H10···F3	0.93	2.52	3.436	170	1–x,1–y,1–z
**3**	C4–H4···F1	0.93	2.60	3.188	159	–x,1–y,1–z
	C8–H8···F2	0.93	2.58	3.133	159	1–x,2–y,–z
**5**	O1–H1A···N1	0.82	1.93	2.743	172	–x,1–y,1–z

**Figure 1:**
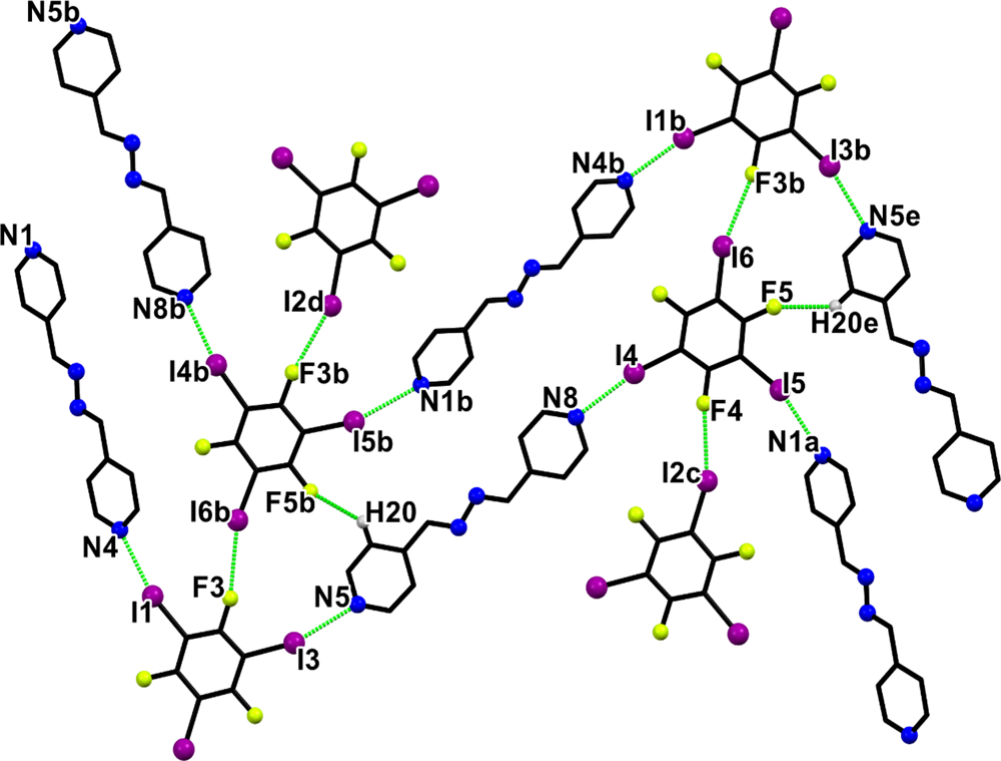
Supramolecular two-dimensional layer in crystal **1**. The symmetry operations a = *x*, −1+*y*, *z*; b = 5/2 – *x*, 1/2 + *y*, 3/2 – *z*; c = −*x*, 1 - *y*, 1 – *z* and d = 5/2 + *x*, 3/2 – *y*, 1/2 + *z* and e = 5/2 – *x*, −1/2 + *y*, 3/2 – *z* generates equivalent atoms. Atoms: carbon (black), nitrogen (blue), iodine (purple), and fluorine (yellow). Hydrogen atoms were omitted for clarity.

To improve the solubility of **1,3,5-titfb**, the cocrystallization was performed in a chloroform–methanol mixture, using the same equivalent ratios. Slow evaporation at room temperature also yielded yellow crystals. However, the X-ray analysis revealed a distinct crystal structure as shown in the crystallographic data presented in Table 2.

This cocrystal, termed **2**, crystallizes in the monoclinic system, belonging to the *P*2_1_/*n* space group. As shown in Figure S5, the I···N halogen bonds are formed, with **1,3,5-titfb** again acting as a bifunctional halogen bond donor and the nitrogen atom serving as the acceptor. The C–I···N interactions are almost linear, with C–I1···N1 and C–I2···N4 angles of 176.9° and 173.9°, respectively, as shown in Table 4. The halogen bonds are 17.3–19.8% shorter than the sum of the van der Waals radii, suggesting significant interaction strength. Additionally, a secondary C–H···F hydrogen bond of 2.52 Å was observed for the C10–H10···F3 interaction at an angle of 170° (Figure S6 and Table 5). A straightforward layered packing arrangement is formed by π—π interactions of parallel-oriented pyridine rings at an approximate distance of 3.85 Å (Table S2), as illustrated in Figure S7. In addition, these sheets are further stabilized through weak F···π­(C=C) (C14–F1··· π; d _F···π_ = 3.407 Å).

To verify the phase purity and homogeneity of the bulk crystalline polymorphs, powder X-ray diffraction (PXRD) data were collected at room temperature and compared to the corresponding simulated patterns. As illustrated in Figures S8–S12, the experimental PXRD patterns of the polymorphs closely match the simulated ones, confirming the high phase purity of the bulk materials. Moreover, the good similarity between the experimental and simulated diffraction profiles indicates that polymorphs **1** and **2** of the (**4-bpdb)·(1,3,5-titfb**) cocrystal share the same structural framework, with minor intensity variations attributed to the preferred orientations of the powdered samples.

Given the observed 1:1 assembly preference between **1,3,5-titfb** and **4-bpdb**, stoichiometric experiments were conducted in a chloroform–methanol mixture. Under these conditions, polymorph **1** was obtained as the major product, accompanied by a new polymorph, designated as **3**. Single-crystal X-ray diffraction analysis of **3** revealed that it crystallizes in the monoclinic *P2_1_/m* space group with the asymmetric unit containing a stoichiometric XB donor and acceptor connected through C–I···N halogen bonds. Similar to **1**, two iodine atoms from **1,3,5-titfb** participate in halogen bonds, C–I1···N1 and C–I3···N3, with pyridine nitrogen atoms of 2.902–2.994 Å (Figure S13). These interactions are characterized by bond angles of 167.0 and 167.1° and bond distances of 2.902 and 2.994 Å, respectively (Table 4). These distances represent a shortening of 17.8 and 15.2% relative to the sum of the van der Waals radii of nitrogen and iodine, indicating relatively strong halogen bonding. Furthermore, a C–I···F halogen bond was also observed, specifically involving the C15–I2···F3 interaction, with a nearly linear angle of 178.9° and a short contact distance of 2.932 Å (Figure S13). These directional interactions contribute to the organization of the halogen-bonded chains, which are further connected to pairs through C–I···F contacts. Additionally, hydrogen bonding interactions were also observed between C4–H4···F1 and C8–H8···F2, with distances of approximately 2.60 Å and an angle of 159° (see Table 5). Unlike cocrystal **1**, in compound **3**, both fluorine atoms are involved in C–H···F interactions, while the third fluorine atom of the **1,3,5-titfb** molecule participates in a halogen···halogen interaction with an iodine atom. In contrast, in **1**, only one fluorine atom is engaged in a nonclassical hydrogen bond, resulting in geometrically distinct cocrystals. In addition, C–I···π and C–F···π interactions also play a role in stabilizing the supramolecular organization of the chains (Figures S14 and S15 and Table S1). The comparison between experimental PXRD patterns and the simulated diffractogram for **3** is in agreement with a mixture of the two polymorphs (Figure S10).

#### Halogen-Bonded Assembly with **1,2-ditfb**

By replacing the XB donor with 1,2-diiodotetrafluorobenzene, the cocrystallization with **4-bpdb** yielded cocrystal **4**. This system crystallizes in the monoclinic *P2_1_/n* space group (Table 3) with a **1,2-ditfb**/**4-bpdb** molar ratio of 2:1. The **1,2-ditfb** and **4-bpdb** molecules are connected via intermolecular C–I···N halogen bonds of 2.866 Å and a bond angle of 175° (Table 4). The nitrogen atom forms shorter halogen bonds, 18.8% shorter than the sum of the van der Waals radii. The second iodine atom is engaged in a halogen···halogen interaction with another iodine atom (d_I···I_ = 3.879 Å, ∠C–I···I = 173.8°), leading to the formation of a discrete tetrameric assembly (Figure 2). A halogen atom can act as a donor, typically displaying a bond angle close to 180°, as observed so far. However, one of the iodine atoms in **1,2-ditfb** also acts as an acceptor, as indicated by a bond angle of approximately 98.5°. According to the literature, when a halogen atom exhibits an angle near 90°, it is considered to be functioning as an acceptor in the interaction.^54^ Moreover, the supramolecular organization of the chains is further reinforced by weak C–I···π and C–F···π interactions (C12–I2··· π, d_I···π_ = 3.968 Å and C09–F2··· π, d_F···π_ = 3.636 Å) similar to that observed in cocrystal **1** (see Supplementary Figure S14 and S16 and Table S1). As presented in Figure S11, the experimental PXRD pattern of cocrystal **4** closely matches the simulated data, confirming the phase purity of the bulk material.

**Figure 2:**
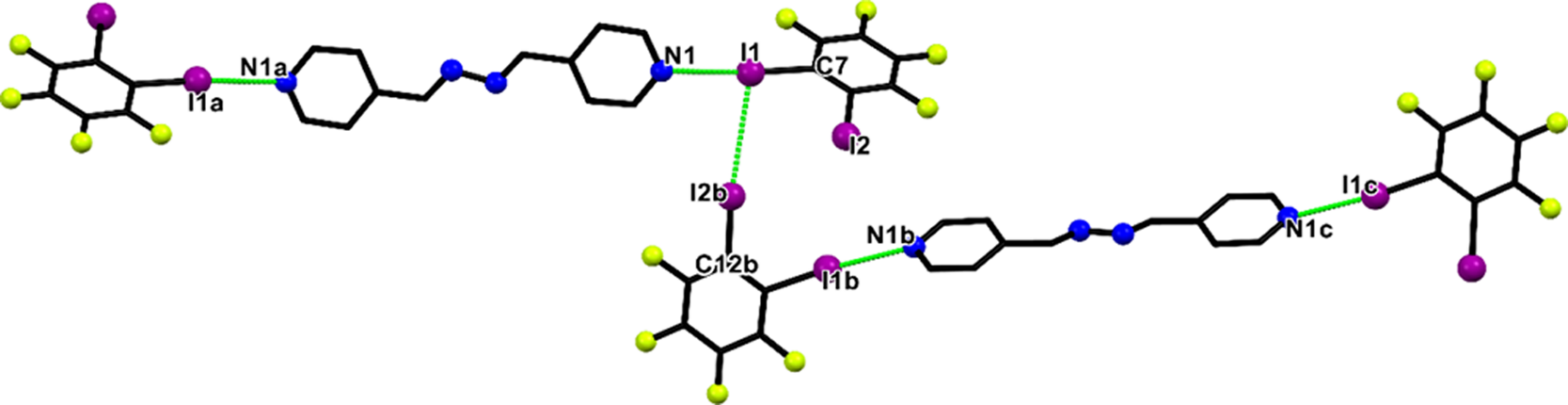
Crystal structure of **4**. The symmetry operations *a* = −*x*, 2–*y*, 2–*z*; *b* = 1–*x*, −1/2+*y*, 3/2–*z*, and *c* = 1+*x*, 3/2–*y*, −1/2+*z* generate equivalent atoms. Atoms: carbon (black), nitrogen (blue), iodine (purple), and fluorine (yellow). Hydrogen atoms were omitted for clarity.

#### Hydrogen-Bonded Assembly with **4,4′-biphenol**

Finally, an investigation was carried out on the use of **4-bpdb** as a hydrogen-bond acceptor in the presence of **4,4′-biphenol** as the donor, yielding cocrystal **5**. It crystallizes in the monoclinic *P*2_1_/*n* space group (Table 3). The molecular structure, including the atom labeling scheme, is shown in Figure 3. The C1–N1–C5 bond angle of 116.98° provides clear evidence that no proton transfer occurs from **4,4′-biphenol** to **4-bpdb**. It is well established that the C–N–C angle in pyridine rings is sensitive to protonation, with the cationic form typically exhibiting larger angles (approximately 121°) compared to its neutral counterpart (around 116°).^55^ The supramolecular aggregates formed between **4,4′-biphenol** and **4-bpdb** consist of heterosynthons, characterized by a hydrogen bond between O1–H1A···N1 of 2.743 Å and a nearly linear angle of 172° (Table 5). Additionally, weak C–H···π interactions were observed between pyridine rings within the layers, involving C4–H4···π and C12–H12···π contacts, with respective distances of 2.95 and 2.91 Å (Figure S17 and Table S1). The experimental PXRD pattern of cocrystal **5** matches well with the simulated data, confirming the phase purity of the bulk material, with a good agreement between the simulated and experimental patterns (Figure S12).

**Figure 3:**
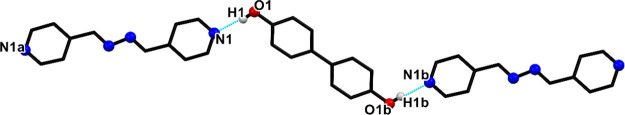
Crystal structure of **5**. The symmetry operations *a* = −*x*, 1–*y*, 1–*z* and *b* = 1–*x*, −*y*, 1–*z* generate equivalent atoms. Atoms: carbon (black), nitrogen (blue), hydrogen (white), and oxygen (red). Nonrelevant hydrogen atoms were omitted for clarity.

### Theoretical Calculations

The main focus of the theoretical approach is the multitude of the halogen interactions (I···N, I···I, I···F) that take place within the synthesized cocrystals. From a multitude of recommended features to characterize these types of intermolecular contacts,^56^ we adopted the following ones:

i) the calculation of the electrophilicity of specific regions on the halogen atoms, namely, the local most maxima (ES*P*_max_ – σ hole);
ii) the calculation of the interaction/binding energy (ΔE_int,XBDA_ = E_XBDA_ – (E_XBD_ + E_XBA_)), which refers to the difference of the sum of the total electronic energy of the halogen-bonded system R-X···Y (XBDA—halogen bond donor–acceptor) and the total energy of the isolated halogen bond donor (XBD) and halogen bond acceptor (XBA) entities. The strengths of the halogen interactions were divided in seven scales of strength:^57^ ultrastrong (above 40 kcal/mol), very strong (between −25 and −40 kcal/mol), strong (between −25 and −15 kcal/mol), moderately strong (between −5 and −15 kcal/mol), weak (between −3 and −5 kcal/mol), very weak (between −1 and −3 kcal/mol), and vdW type (between −0.01 and −1 kcal/mol). Therefore, for this purpose, the halogen bond donor–acceptor complexes (XBDA) were isolated from the X-ray crystallographic measurements;
iii) the calculation of the orbital–orbital stabilization energy by the second-order perturbation theory based on using natural bond orbital analysis (NBO).^58,59^ This is because some charge transfer might occur between the frontier XBA orbital (filled lone pair orbital lp) and the XBD frontier (empty σ*/π* type antibonding orbital); and
iv) by the aid of quantum theory of atoms in molecule (QTAIM),^60^ important topological parameters such as electron densities (ρ_BCP_) between XBD and XBA, sign and magnitude of the Laplacian (∇_BCP_^2^), and the total energy density at bond critical points (*H*_BCP_) were derived to characterize the interactions. Bader’s QTAIM theory has been recognized as a very successful theory for analyzing the physical nature of intermolecular interactions.^61^

Part of the abovementioned methods (Δ*E*_int_, NBO, and QTAIM approaches) are also used for the description of the hydrogen contacts or for stacking interactions.

Despite the fact that the iodo-fluorobenzene structures are intensively used as important halogen bond donors in crystal engineering and were studied intensively from various perspectives,^62^ we analyze these two polytopic halogen bond donors **1,2-ditfb** and **1,3,5-titfb** from the perspective of the newly synthesized compounds.

To ascertain differences in behavior of the two iodo-floro molecules as XBDs in **1**, **2**, **3** and **4** cocrystals, the series of quantum chemical calculations were aimed at observing the differences in their interactions with the azine molecule. The value of the ES*P*_max_ for the two iodo-fluoro molecules is 32.15 kcal/mol for the **1,2-ditfb** molecule, slightly more positive compared to the **1,3,5-titfb**, which is 31.5 kcal/mol. Similar values were reported for these molecules at M06–2X/def2-TZVPP level (30 kcal/mol for the **1,3,5-titfb** and 32 kcal/mol for 1,4 diiodo-2,3,5,6-tetrafluoror molecules).^63^ The supplementary test with M06–2X/def2-TZVPP level of theory was carried out and a value of ES*P*_max_ = 30.43 kcal/mol was obtained for **1,3,5-titfb**, thus very close to the values obtained at ωB97XD/def2-TZVPP level and to those obtained in literature at the same level of theory.

The computations on the I···N systems have shown for all four compounds, that the interaction between the single **4-bpdb** molecule with one of the iodine atoms of the **1,3,5-titfb** and **1,2-ditfb** molecules reduces the ES*P*_max_; on the unbonded iodine atom(s) by 4.52, 3.89 in **1**; and by 6.95, 5.04, and 5.04 kcal/mol in **2**, **3**, and **4** (Figure 4). This represents a significant decrease but still gives the possibility of forming the second halogen interaction with the second unbonded iodine. In crystals **1**, **2**, and **3**, it forms the second N···I contact (I3···N5 and I5···N1 in **1**, I5–N1 in **2**, and I3–N3 in **3**), while in **4**, it forms the I···I halogen bonding. This reduction of ES*P*_max_ is mirrored in the longer N···I contact distances (see Table 4) and accordingly to the decrease of the interaction energies (Δ*E*_int,XBDA_). This second N···I interaction reduces further the ESP_max_ on the third remaining unbonded iodine atom by 3.92 and 4.08 kcal/mol in **1** and by 5.04 and 3.78 kcal/mol in **2** and **3.** The binding/interaction energy of the first **4-bpdb** molecule with the first iodine of **1,3,5-titfb** molecule in **1** (N4···I1/N8···I4), **2** (N1···I1), **3** (N1···I1), and **4** (N1···I1) is −5.47, −6.05, −6.14, −6.15, and −5.56 kcal/mol, while binding of the second **4-bpdb** molecule with the second unbonded iodine atom in the **1,3,5-titfb-4-bpdb** dimer in **1** (N5···I3/I5···N1), **2** (I2···N4), and **3** (I3···N3) is −4.73, −4.98, −5.63, and −5.33 kcal/mol. Similar effects of binding N based molecules on these polytopic XBD were recently reported.^62^ This reveals how electron densities in both the donor and the acceptor molecules are perturbed by formation of the halogen bond. This increase of electron density is not observed only in the σ hole region of the iodine atoms but is coupled with an increase of electron density perpendicular to it or of the fluorine atoms. This increase of the electron density on fluorine atoms is marked on all atoms in Figure 4. ESP_min,F_ increases with approximately 3.5 to 6 kcal/mol when the first N···I contact is formed and with ≈8.5 to 9.5 kcal/mol when the second contact is formed. The increase of the electron densities on fluorine atoms explains the formation of the −C-H···F and also of the I···F contacts. It also explains the formation of the type II I ···I contacts in **4**, by the increase in the electron density perpendicular to the σ-hole.

**Figure 4:**
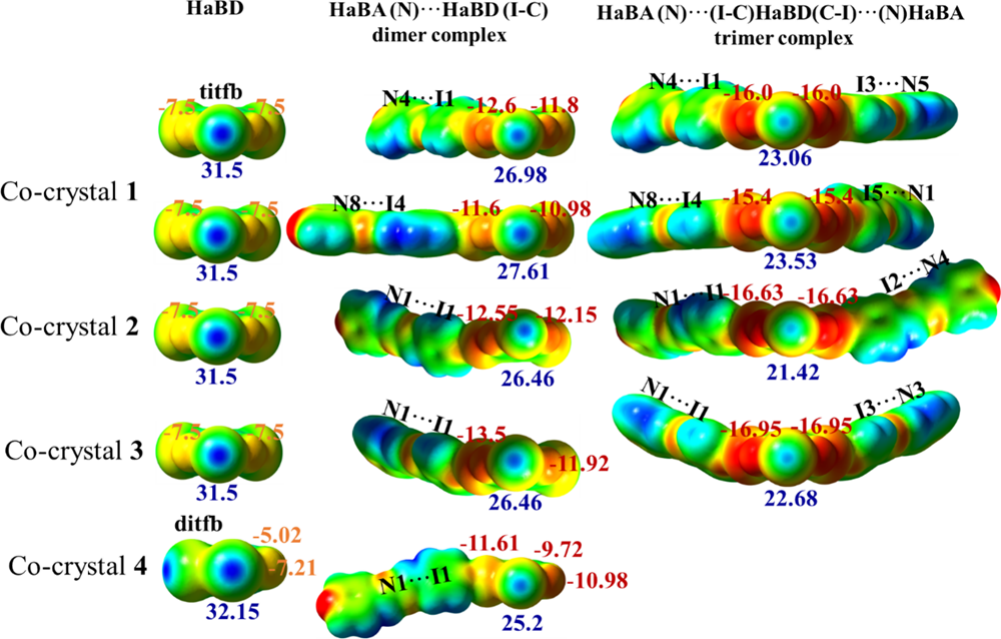
ESP mapped on the electron density isosurface (ρ_el_ = 0.001 au) of XBD in cocrystals **1**, **2**, and **3** (in **1,3,5 titfb**, **1,3,5-titfb**·**4-bpdb**, and **1,3,5-ditb**-(**4-bpdb**)) and in cocrystal **4** (in **1,2-ditfb**, **1,2-ditfb**·**4-bpdb**). Boundaries of ESP are (−0.03 au (red) and 0.05 au (blue region)). The σ-hole magnitude (ESP_max,I_-blue) of iodine and the minimum magnitude on fluorine (ESP_min,F_-orange on **1,3,5-titfb** and **1,2-ditfb** molecules/red on 1:1 and 1:2 systems). Values are given in kcal/mol.

According to the strength of the halogen bond classification, with the exception of the N5···I3 and I5–N1 interactions that are in the “weak interaction” region but close to the upper limit, all the other values are in the “moderately strong” region, at their turn close to the low limit of the domain.

From the QTAIM perspective at I···N intermolecular bond critical points (BCPs), all four systems present typical properties of closed-shell interactions (Figure 5 and Figure S18 and Table S3). This is because the value of electron density (ρ_BCP_) shows small values and the Laplacian of electron density ∇_BCP_^2^ ρ is positive. Correlated with the positive value of *H*_BCP_ excludes a covalency or partial covalency character of these interactions. The electron density values range from 0.014 to 0.02 au, and the Laplacian of the electron density ranges from 0.058 to 0.095 au. Similar values were reported for systems that form the I···N contacts in PhF5I···NMe3/PhF5I···N-Pyr/PhFI5···NH3 but obtained at ρ_BCP_ = 0.0199/0.0159/0.0145 au at M06–2X/def2tzvp level of theory.^64^ In the supplementary tests for the I1···N4 contact in compound **1** at the M06–2X/def2tzvp level of theory, a value of ρ = 0.01903 au was obtained. This validates the similar values with these two levels of theories (ωB97XD/def2-TZVPP and M06–2X/def2-TZVPP or M06–2X/def2tzvp).

**Figure 5:**
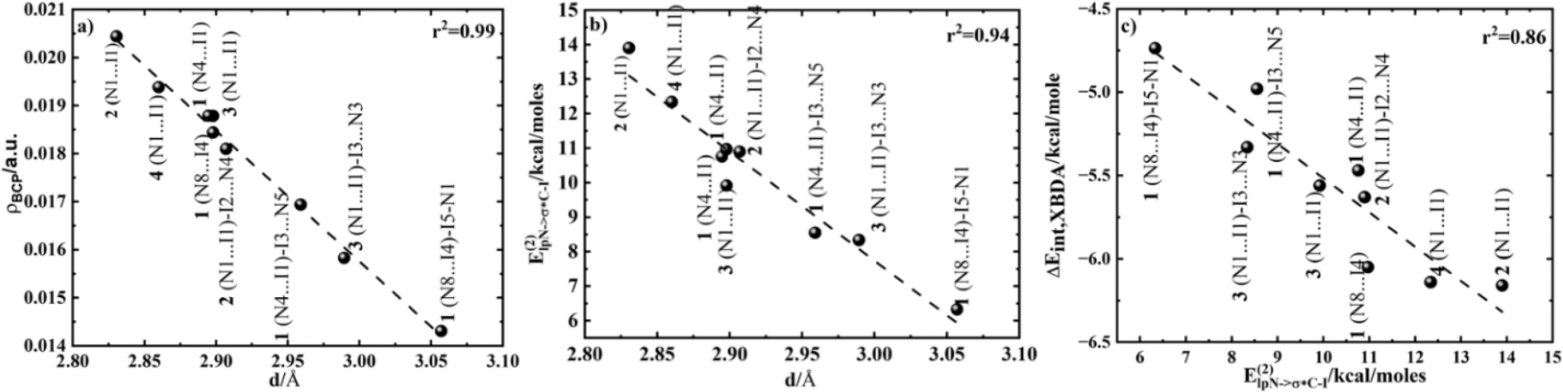
Linear relationships between (a) electron density at BCP (ρ_BCP,I···N_) vs I···N intermolecular distance (d_I···N_); (b) second-order perturbation energy vs I···N intermolecular distance (E^(2)^_lppN→σ*C–I_ vs d_I···N_); (c) interaction energy vs. second order perturbation energy (ΔE_int, XBDA_ vs E^(2)^_lppN→σ*C–I_).

The XBDA complex formation is associated also with the orbital interaction between the lone pair of the electron donor (lp_p,N_) of XBA and the antibonding orbital of the electron acceptor (σ*_C–I_) of XBD. The energy of orbital–orbital interaction estimated by the E^(2)^ second-order perturbation theory is displayed in Table S3 of the Supporting Information for all formed N···I in dimers and trimers. The highest stabilizing energies are attained for the smallest distance contacts. The electron density at bcp and the bond distance in the XBDA complexes present a linear relationship (ρ_BCP,I···N_ vs d_I···N_), with highest electron densities that occur at the smallest distances. Similar linear relationships are attained between the stabilizing energies obtained by the interaction between the lone pair orbitals of nitrogen and the antibonding orbital of the C–I bond and the bond distances (E^(2)^_lppN→σ*(c‑I)_ vs d_I···N_), with the highest stabilizing energy for the smallest distance. Another good correlation was obtained between the same stabilizing energies and the interaction energies (E^(2)^_lppN→σ*(c‑I)_ vs ΔE_int,XBDA_), with stronger interaction energies as the stabilizing energy increases.

The other types of formed halogen interactions in these cocrystals are the I···I and I···F contacts. The I···I halogen interactions in compound **4** are of type II, and both iodine atoms of **1,2-ditfb** are involved in these interactions, one as XBD and the other one as XBA. Therefore, the iodine atoms that act as XBD in the N···I contact act as XBA in the I···I halogen interactions. When acting as XBA, in the dimer I···I complex, the ESP_max_ increases with 3.13 kcal/mol (see dimer complex in Figure 6b) compared to the case when no contacts are formed. When the other iodine atom acts as an XBD in the same I···I interaction (see the trimer in Figure 6c), the ESP_max_ decreases slightly with 0.63 kcal/mol compared to the dimer complex in Figure 6b. Analyzing the dimer complex from the perspective of ES*P*_max_ of the unbonded iodine atoms, it decreases or increases only with ≈1 kcal/mol, when the bonded iodine atom acts as XBD (Figure 6d) or as XBA (Figure 6e), respectively. Clearly, the influence of this I···I interaction on either bonded or unbonded iodine is not as significant as in the case of I···N halogen bonding.

**Figure 6:**
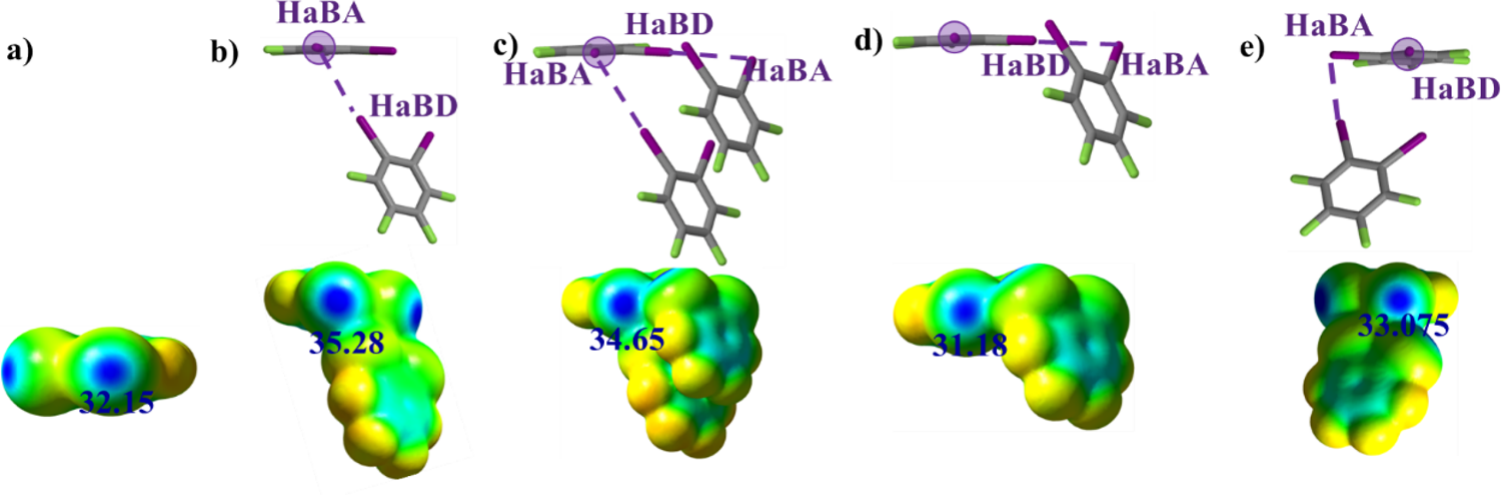
ESP mapped on the electron density isosurface (ρ_el_ = 0.001 au) in **1,2-ditfb** monomer, dimer, and trimer complexes forming I···I contacts in **4** (a) ESP_max_ of iodine in **1,2-ditfb** molecule; (b) ESP_max_ of I1­(XBA) in I1­(XBA) ··I­(XBD) dimer; (c) ESP_max_ of I1 XBA in I1­(XBA)···I­(XBD)/I_2_(XBD)···I (XBA) trimer complex; (d) ESP_max_ of unbonded iodine in I2­(XBD) ···I­(XBA) dimer complex; (e) ESP_s,max_–unbonded iodine in I2­(XBA) ···I­(XBD) dimer complex. Boundaries of ESP are −0.03 (red) and 0.05 (blue region). The σ-hole magnitude (ESP_max_) for each system is given in kcal/mol. The transparent circle indicates the corresponding iodine atom for which ESP_max_ is indicated.

Analyzing the QTAIM indicators (the BP and BCP in Figure S18 and the QTAIM parameters in Table S3, of the Supplementary File) at BCP, they have the same order of magnitude with those reported for the type II contacts in iodo-benzene complex dimers (e.g., ρ = 0.00713 au at MP2/aug-cc-pvdz level of theory).^65^ In terms of orbital–orbital interactions, the stabilizing energies obtained by the same lone pair interactions with the antibonding orbitals are weaker than those obtained for the N···I interactions (e.g., E^(2)^_lppI→σ*(C–I)_ = 3.4 kcal/mol). These values mirror also in the values of the interaction energy in this I···I dimer complex that is Δ*E*_int,I···I_ = −3.3 kcal/mol, and it classifies this halogen bonding as a “weak” one (between or equal to −3 and/or −5 kcal/mol), definitely weaker than I···N bonding.

The I···F contacts, a hetero halogen–halogen bond, which occurs in compounds **1** and **3**, are the weakest halogen–halogen interactions. They take place between the positive region of the σ hole of the third unbonded I atom of the **1,3,5-titfb** molecule and the negative F atom of the second **1,3,5-titfb** molecule. These contacts are of unconventional halogen contacts. Therefore, in cocrystals **1** and **3**, **1,3,5-titfb** acts as a tritopic donor by forming two I···N halogen interactions and one F···I halogen–halogen interaction. According to the calculated interaction energies, they are classified as “very weak” ones (Δ*E*_int,I···F_ = −1.25 to −1.52 kcal/mol) with values closer to the low limit of the interval with the “van der Waals” class. According to NBO analysis, the orbital–orbital interactions are very weak and occur between the lone pair orbitals of fluorine and the antibonding orbitals of C–I bonds and between the lone pair orbitals of iodine and the antibonding orbital of C–F bonds (see in Table S3).

Besides the halogen bond interactions, as a reference, we considered also the hydrogen bond and the π–π stacking interactions that play a vital role in the crystal packing. The −O-H···N hydrogen-bond forms in **5** compound and has an interaction energy of Δ*E*_int,–O‑H···N_ of −6.99 kcal/mol, slightly stronger than those obtained for shorter N···I halogen interactions in **1, 2**, **3**, and **4** compounds. According to the classification of the strength of this hydrogen bonding, it is placed in the “weak to moderate” region (the binding energy in between −2.5 and −14 kcal/mol).^66^ The F···H are in the “very weak” region, being characterized by electrostatic and long-range dispersions.

The investigated stackings are those formed between two **1,3,5-titfb** molecules in **1**, between two **1,2-ditfb** molecules in **4**, and between the **4-bpdb** molecules in the **1**, **2**, and **3** structures. In the stacking between **1,3,5-titfb** molecules, seven bond paths were identified by QTAIM analysis, which are of I···I, C···I, and C···C type, while in the stacking of **1,2-ditfb**, five bond paths were identified by the QTAIM method (see Supplementary File, Figure S18), which are of C···C, C···F, C···I, and F···I types. Small electron densities correspond to each bond critical point, ranging between 0.0015 and 0.0035 au. The interaction energies that include all contacts are −9.24 and −7.61 kcal/mol, therefore a slightly stronger interaction compared to the N···I. For the other three investigated stackings that imply the **4-bpdb** molecule, six, seven, and eight bond paths were identified (see Supplementary File, Figure S18). They are of C···C, N···N, and N···C types with corresponding small values of the electron densities for each contact, which spans between 0.001 and 0.0035 au. Both pyridyl and azine N atoms are engaged in the interaction.

The NBO analysis indicates very weak interactions between the π bonding orbitals of the C–C bond of the ring and the π* antibonding orbitals of the azine N–C bond. Besides this, in this π-π interaction participates also the π bonding orbital of the C–C bond of the pyridyl ring and the π* antibonding orbital of the C–N or C–C bond of the other pyridyl ring (see in Table S3). The corresponding π-π interaction energies of −9.51, −10.14, and −8.84 kcal/mol, as expected, indicate their significant contribution to the crystal packing.

## Final Remarks

The cocrystallization of bis-pyridyl-azine (**4-bpdb**) with various halogen and hydrogen-bond donors afforded three new cocrystals with one of them as three polymorphs. The stoichiometry and crystallization conditions significantly influenced the supramolecular architectures, with **1,3,5-titfb** acting as a versatile halogen bond donor, forming diverse C–I···N and C–I···F interactions. In compound **1**, a 1:1 assembly features zigzag C–I···N chains connected via C–I···F contacts. Changing the solvent mixture, a new polymorph is obtained, which exhibits distinct packing arrangements involving halogen and hydrogen bonding, π–π interactions, and F···π contacts. When **1,2-ditfb** was used instead of **1,3,5-titfb**, cocrystal **4** formed a discrete tetrameric assembly stabilized by I···N and I···I halogen bonds. In contrast, cocrystal **5**, formed with **4,4′-biphenol**, features hydrogen-bonded heterosynthons between phenolic OH groups and pyridine nitrogen atoms.

As previously reported, polyiodofluorobenzenes frequently employ two of their iodine atoms in directional halogen bonding, not solely due to electronic activation but also because this arrangement supports more efficient packing motifs. The same packing-driven tendency is reflected in our systems: cocrystals **1**–**3** exhibit secondary N···I contacts, and, in cocrystals **1** and **3**, the third iodine atom preferentially engages in I···F interactions, whereas in compound **4**, the second iodine forms I···I contacts.

DFT calculations indicate that the formation of N···I halogen bonds may contribute to this behavior by modulating the electrostatic potential of the donor molecules. Coordination of iodine atoms to **4-bpdb** lowers the ESP_max_ values of the remaining free iodine atoms and increases the local electronegativity of the fluorine atoms. The electronegativity increase of fluorine atoms favors the formation of the I···F halogen and of the C–H···F contacts. Notably, in cocrystals **1**–**3**, secondary N···I halogen bonds are established while the third I atoms of **1** and **3** form I···F halogen bonds. In compound **4**, the uncoordinated iodine forms an I···I contacts.

Overall, these findings highlight that subtle electronic modulations introduced by the **4-bpdb** donor operate in concert with strong packing-symmetry preferences intrinsic to polyiodinated fluorobenzenes. The resulting balance between electronic effects and packing efficiency ultimately guides the formation of the distinct supramolecular motifs obtained.

## Supplementary Material



## Notes

^1^H NMR spectrum of compound **4-bpdb** (Figure S1); partial expanded ^1^H NMR spectrum of compound **4-bpdb** (Figure S2); crystallization diagrams of cocrystals **1-5** (Figures S3-7 and Figures S13-17); simulated and experimental PXRD for **1-5** (Figures S8-12); molecular graphs of stacking in **1-4** extracted from the corresponding crystals (Figure S18); geometric parameters of C–X···π interactions in cocrystals **1**–**5** (Table S1); π-π stacking interactions in co-crystal **2** (Table S2); local properties obtained from QTAIM analysis method (Table S3) (PDF)
