# Supplementary material for: Engineering Supramolecular Systems with a Bis(pyridyl)azine Derivative and Different Hydrogen and Halogen Donors
Source: Cryst Growth Des. 2025 Dec 26;26(1):198–209. doi: 10.1021/acs.cgd.5c01135 (PMC12787669; doi:10.1021/acs.cgd.5c01135)
Supplement: Supplementary file 1 [file cg5c01135_si_001.pdf]

Supplementary Information

Engineering Supramolecular Systems with a Bis(pyridyl)azine Derivative and Different Hydrogen and Halogen Donors

Mayra S. Coutinho[1,2,a], Thomaz de A. Costa[2,a], Alan Imperatori[2], Andrei A. Patrascu[3,4], Isabela Man[3], Maria G. F. Vaz[2], Simona Nica[4], Marius Andruh[3,4], Pedro N. Batalha[2]

[1] *Chemistry Department, Instituto Federal do Paraná, Palmas, PR, Brasil.*
[2] *Instituto de Química, Universidade Federal Fluminense, Centro, Niterói, RJ, Brasil.*
[3] *Inorganic Chemistry Laboratory, Faculty of Chemistry, University of Bucharest, Bd. Regina Elisabeta 4-12, 030018-Bucharest, Romania;*
[4] *"C. D. Nenitzescu" Institute of Organic and Supramolecular Chemistry of the Romanian Academy, Splaiul Independentei 202 B, 060023-Bucuarest, Romania;*

[a] *These authors contributed equally to this work.*
**Corresponding authors:*
Simona Nica: simona.nica@icoscdn.ro;
Marius Andruh: marius.andruh@acad.ro;
Pedro Netto Batalha: pedrobatalha@id.uff.br

Contents

Supplementary Figures	2
Supplementary Tables	6

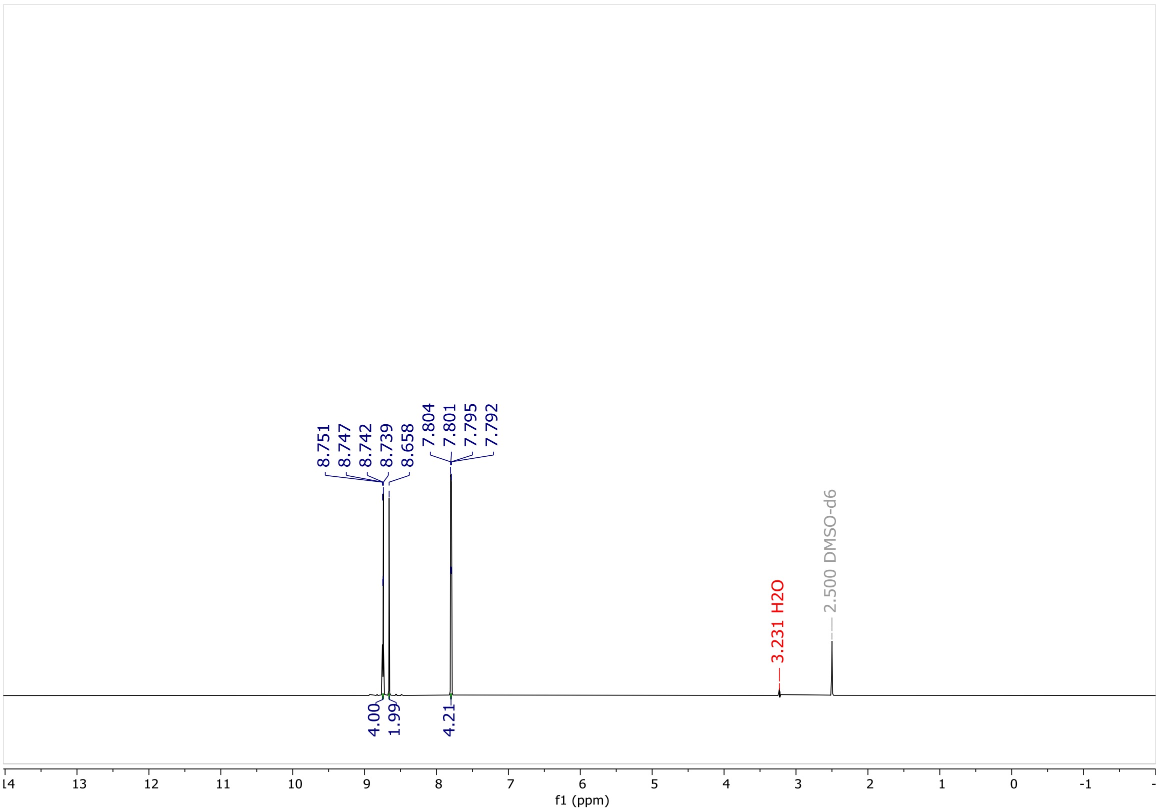

Figure S1: ^{1}H NMR spectrum of compound **4-bpdb** (500.00 MHz, DMSO-d_6).

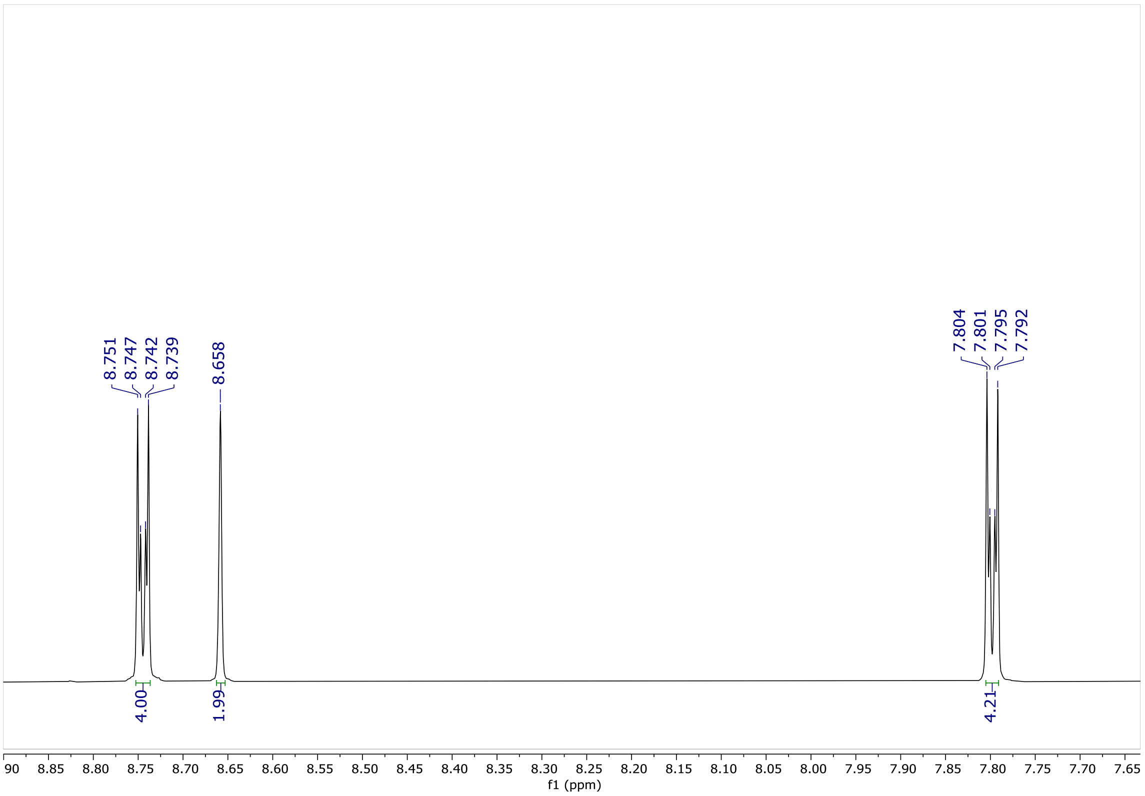

Figure S2: Expanded ^{1}H NMR spectrum of compound **4-bpdb** (500.00 MHz, DMSO-d_6).

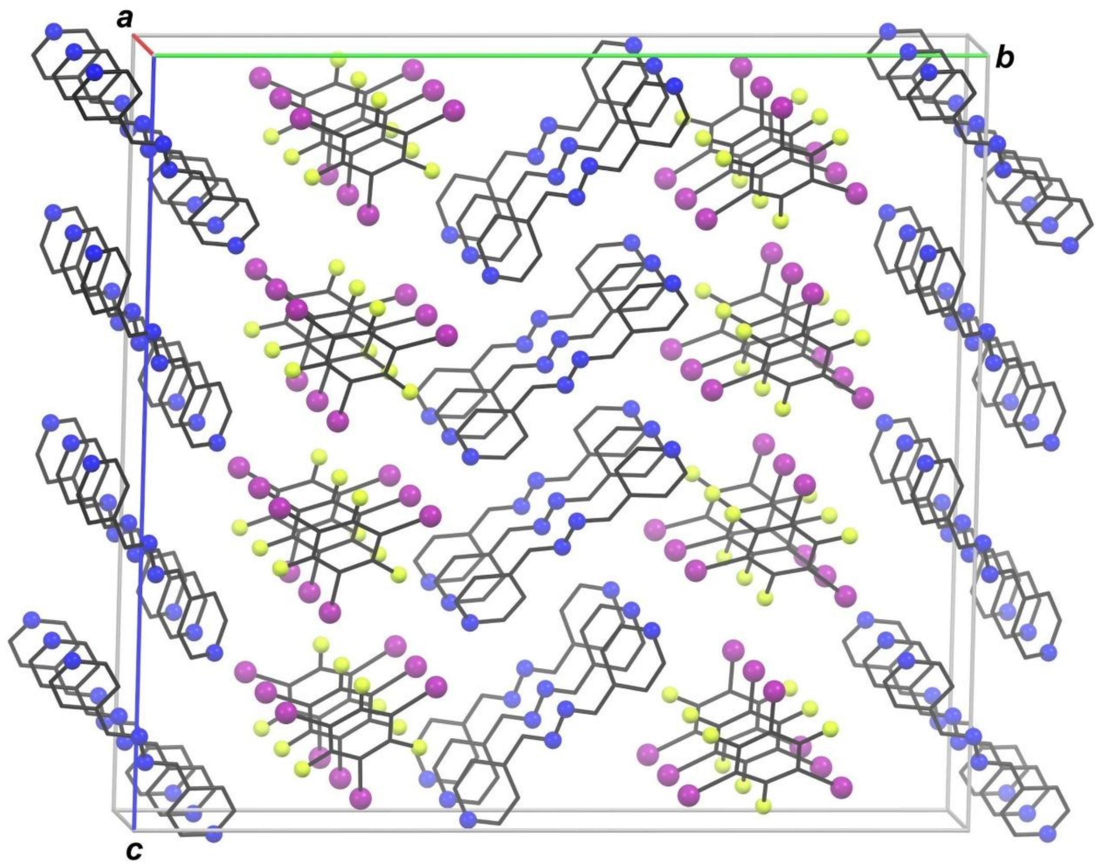

Figure S3: Packing diagram of co-crystal **1**

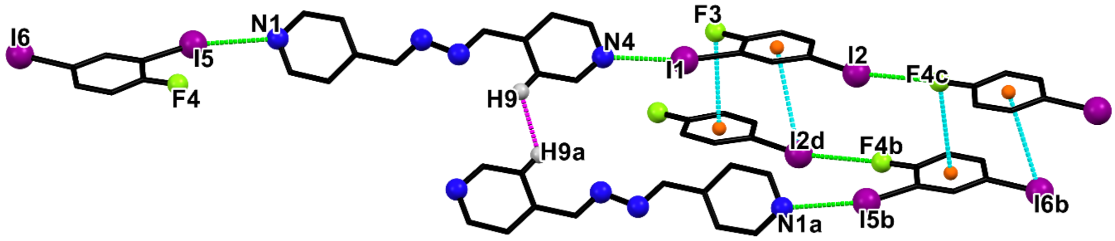

Figure S4: C–X···π (cyan) and C-H···H-C (magenta) interactions in co-crystal **1**.The symmetry operations a = 1-x, 2-y, 1-z; b = 1-x, 1-y, 1-z; c = -x, 1-y, 1-z; d = 1+x, y, z. Atoms: carbon (black), nitrogen (blue), iodine (purple), and fluorine (yellow). Non-relevant hydrogen, fluorine and iodine atoms were omitted for clarity.

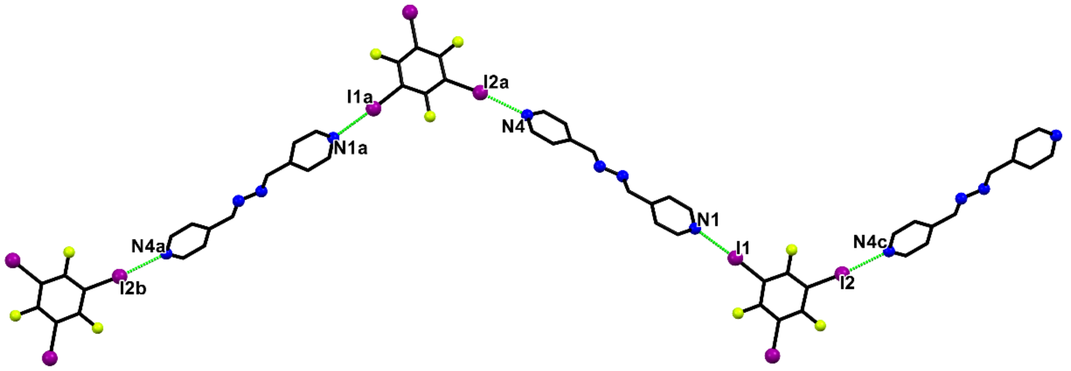

Figure S5: Crystal structure of **2**. The symmetry operations a = -3/2 + *x*, 3/2 - *y*, ½ + *z*; b = -3 + *x*, y, 1 + *z* and c = 3/2 + *x*, 3/2 - *y*, -1/2 + *z* generates equivalent atoms. Atoms: carbon (black), nitrogen (blue), iodine (purple), and fluorine (yellow). Hydrogen atoms were omitted for clarity.

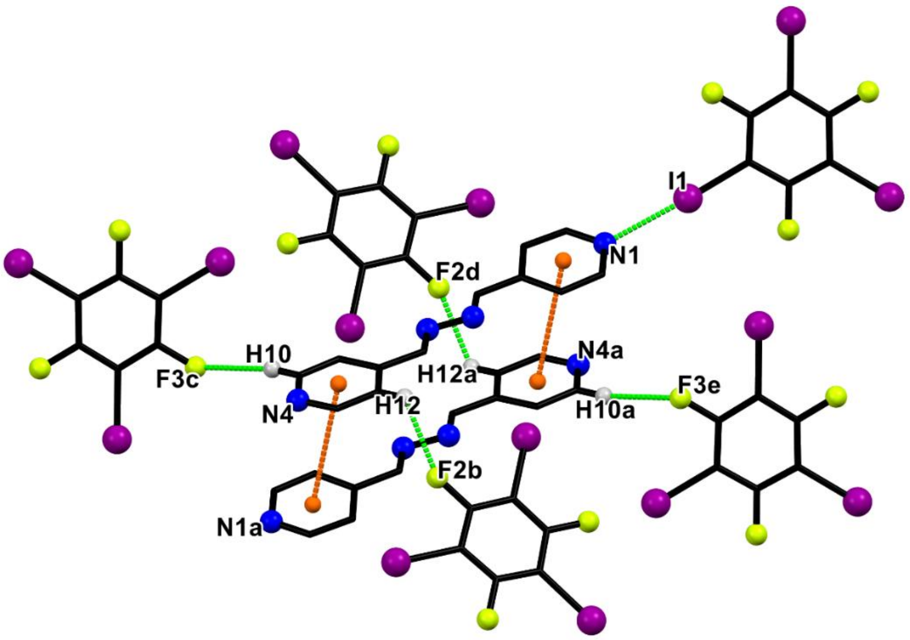

Figure S6: Non-conventional C–H···F hydrogen bond in crystal **2**. The symmetry operations: a = 1 - *x*, 2 - *y*, 1 - *z*; b = 3/2 - *x*, 3/2 + *y*, ½ - *z*; c = 1 - *x*, 1 - *y*, 1 - *z*; d = -1/2 + *x*, ½ - *y*, ½ + *z* and e = *x*, 1 + *y*, *z* generates equivalent atoms. Atoms: carbon (black), nitrogen (blue), iodine (purple), and fluorine (yellow). Non-relevant hydrogen atoms were omitted for clarity.

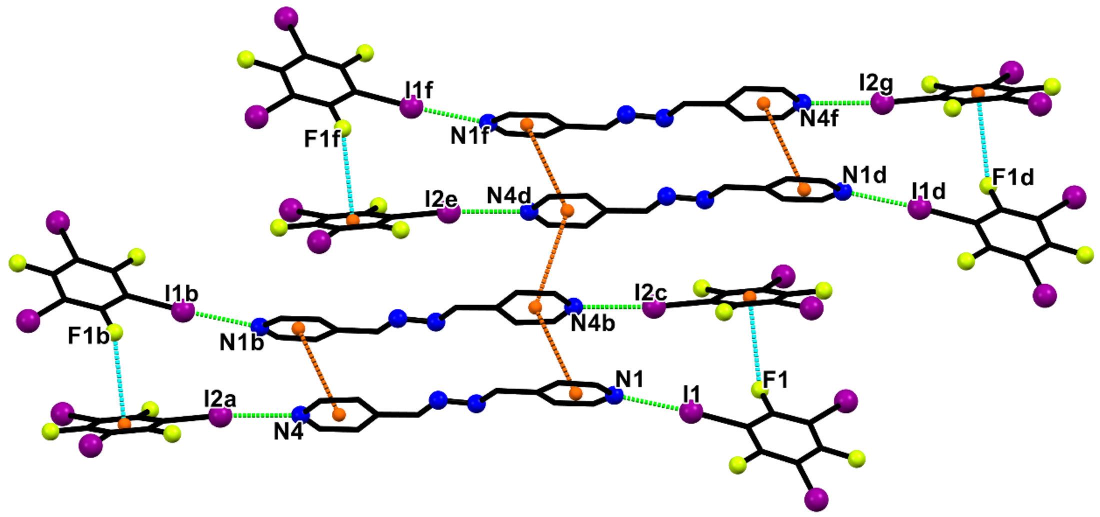

Figure S7: F···π(C═C) and π–π stacking interactions in crystal **2**. The symmetry operations a = -3/2 + *x*, 3/2 - *y*, ½ + *z*; b = 1 - *x*, 2 - *y*, 1 - *z*; c = 5/2 - *x*, ½ + *y*, ½ - *z*; d = 1 + *x*, *y*, *z*; e = - ½ + *x*, 3/2 - *y*, ½ + *z*; f = 2 - *x*, 2 - *y*, 1 - *z* and g = 7/2 - *x*, ½ + *y*, ½ - *z* generates equivalent atoms. Atoms: carbon (black), nitrogen (blue), iodine (purple), and fluorine (yellow). Hydrogen atoms were omitted for clarity.

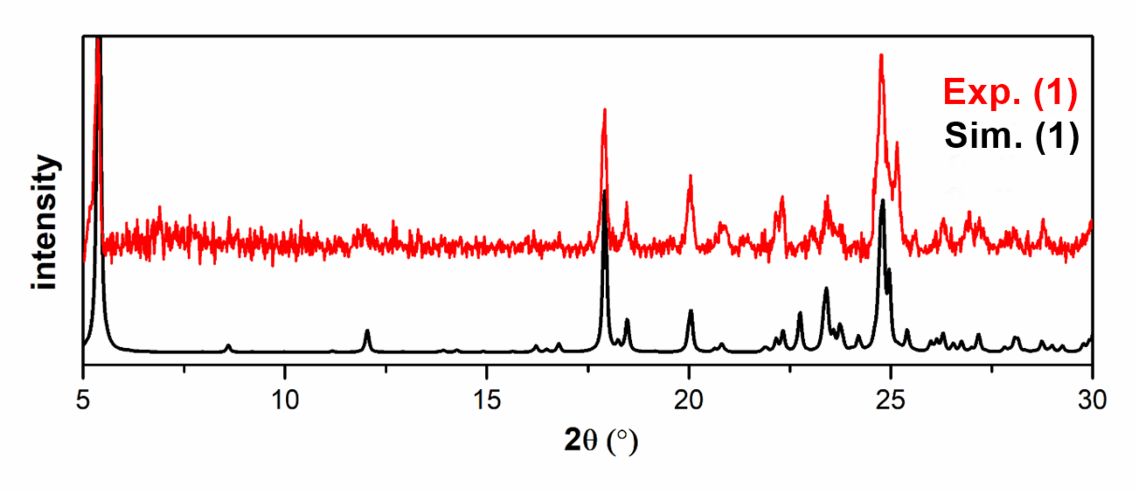

Figure S8 - Simulated and experimental powder X-ray diffractograms for **1**

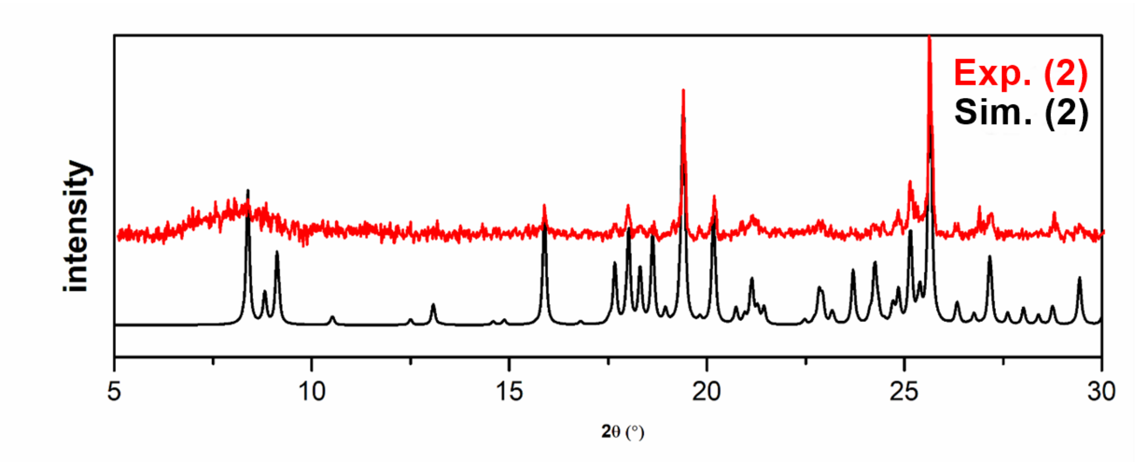

Figure S9 - Simulated and experimental powder X-ray diffractograms for **2**

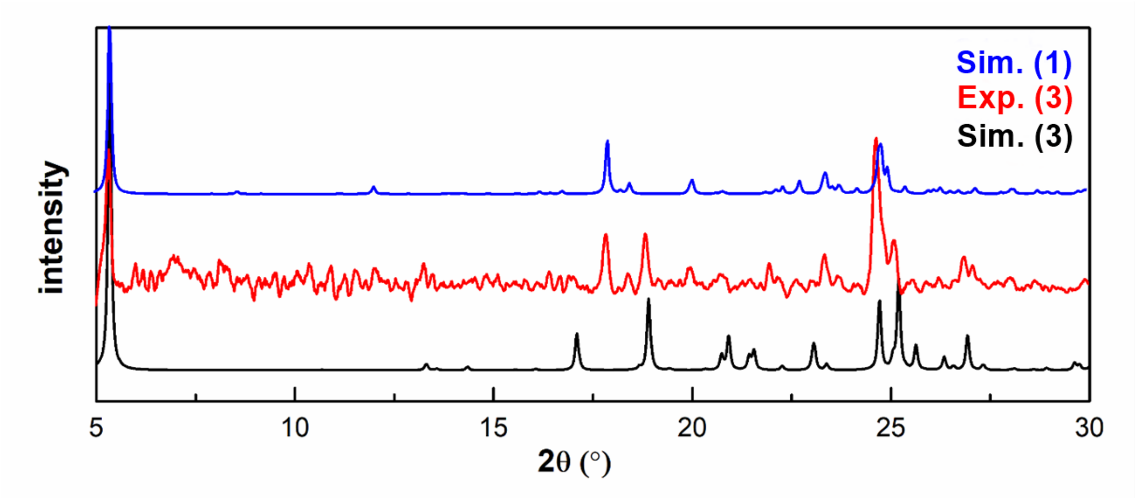

Figure S10 - Simulated and experimental powder X-ray diffractograms for **3**

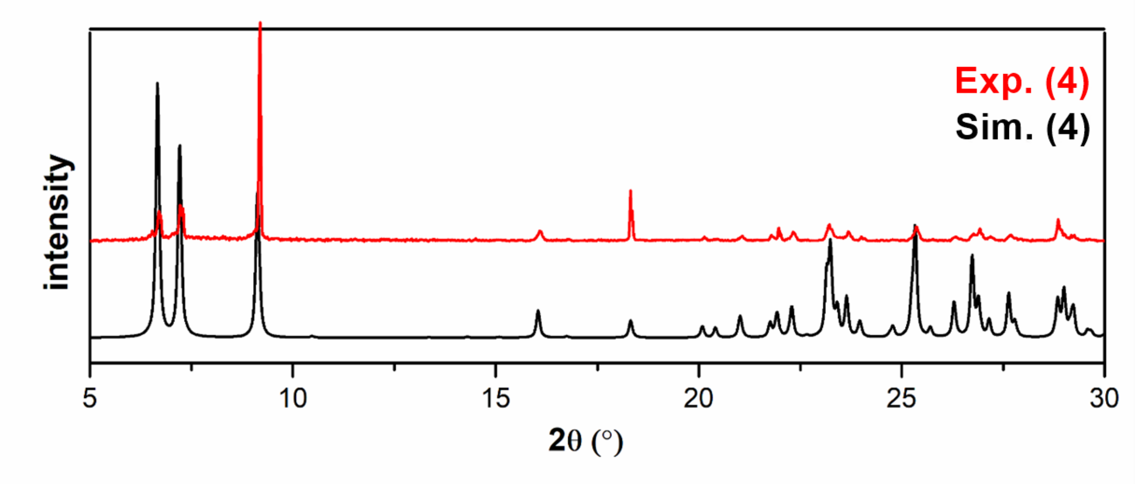

Figure S11 - Simulated and experimental powder X-ray diffractograms for **4**

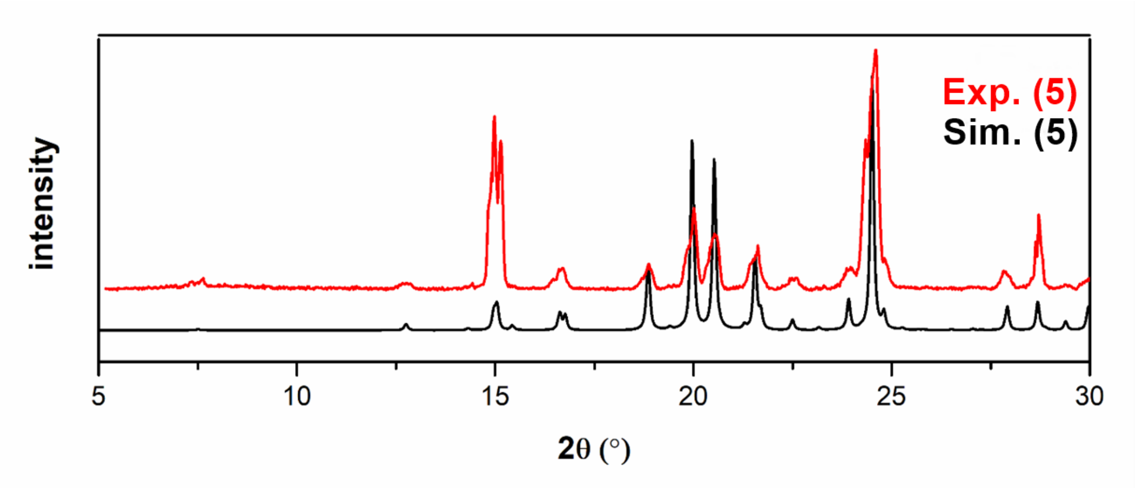

Figure S12 - Simulated and experimental powder X-ray diffractograms for **5**

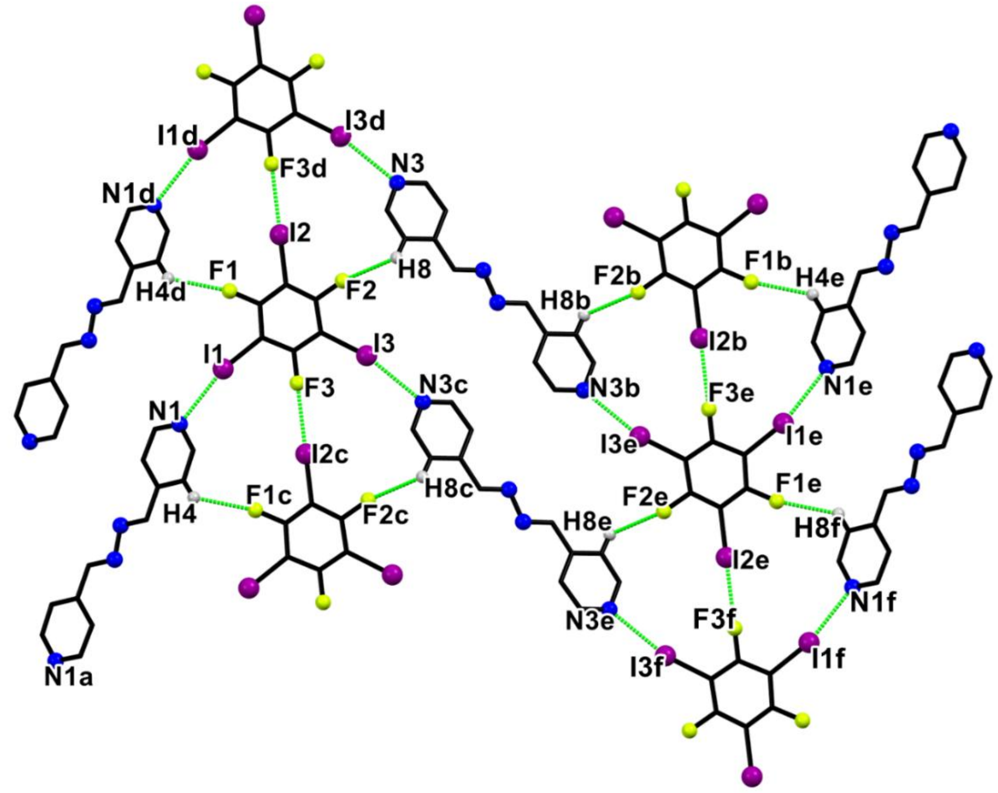

Figure S13: Supramolecular two-dimensional layer in crystal **3**. The symmetry operations a = -1-*x*, -*y*, 1-*z*; b = 1-*x*, 2-*y*, -*z*; c = -1+*x*, -1+*y*, *z*; d = 1+*x*, 1+**y**, *z*; e = -*x*, 1-*y*, -*z* and f = -1-*x*, -*y*, -*z* generates equivalent atoms. Atoms: carbon (black), nitrogen (blue), iodine (purple), and fluorine (yellow). Non-relevant hydrogen atoms were omitted for clarity.

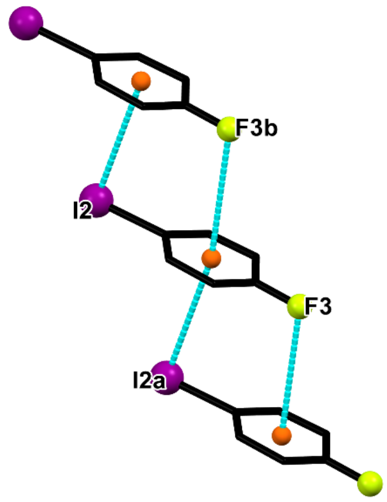

Figure S14: Illustration of the general geometry of C–I···π and C–F···π interactions in co-crystal **1**. The symmetry operations a = 1+x, y, z and b = -1+x, y, z

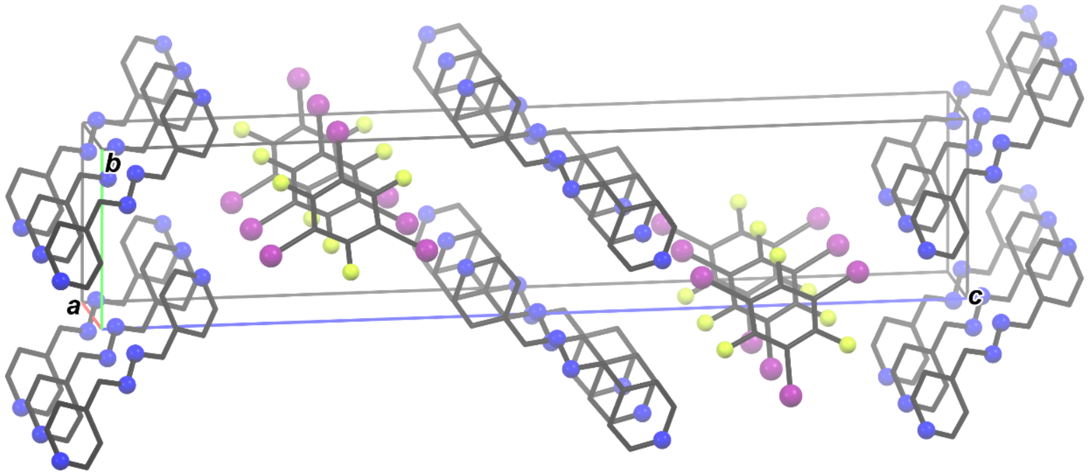

Figure S15: Packing diagram of co-crystal **3.**

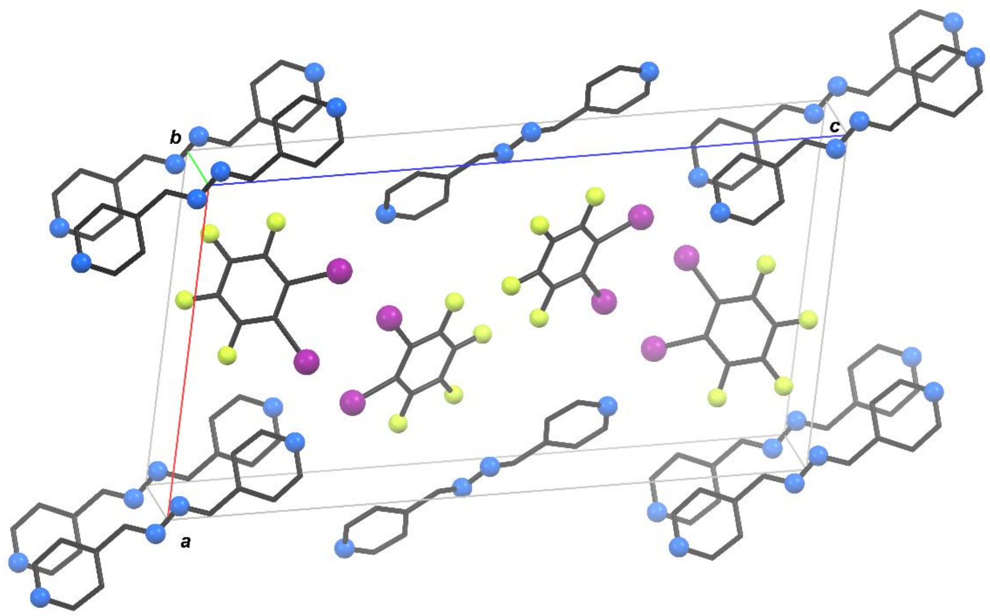

Figure S16: Packing diagram of co-crystal **4.**

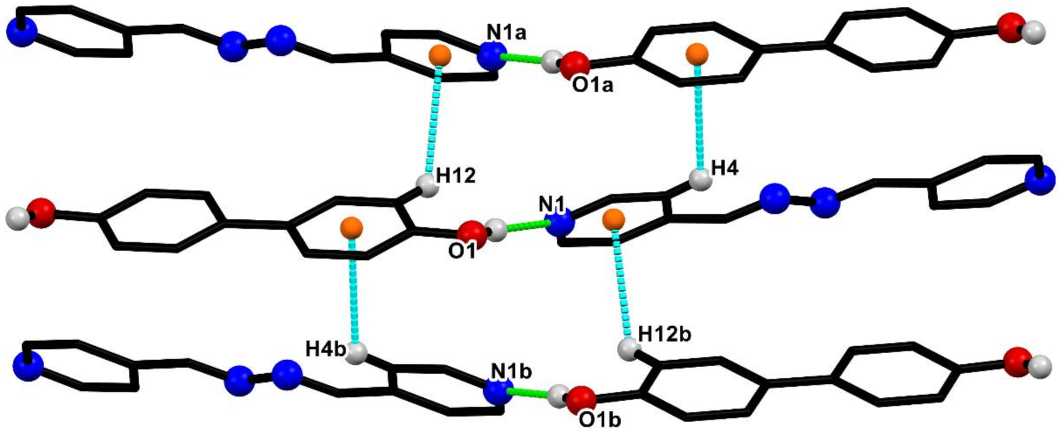

Figure S17: C–H···π interactions of the co-crystal **5**. The symmetry operations a = x, 1/2-y, 1/2+z and b = x, 1/2-y, -1/2+z generates equivalent atoms. Atoms: carbon (black), nitrogen (blue), hydrogen (white) and oxygen (red). Non-relevant hydrogen atoms were omitted for clarity.

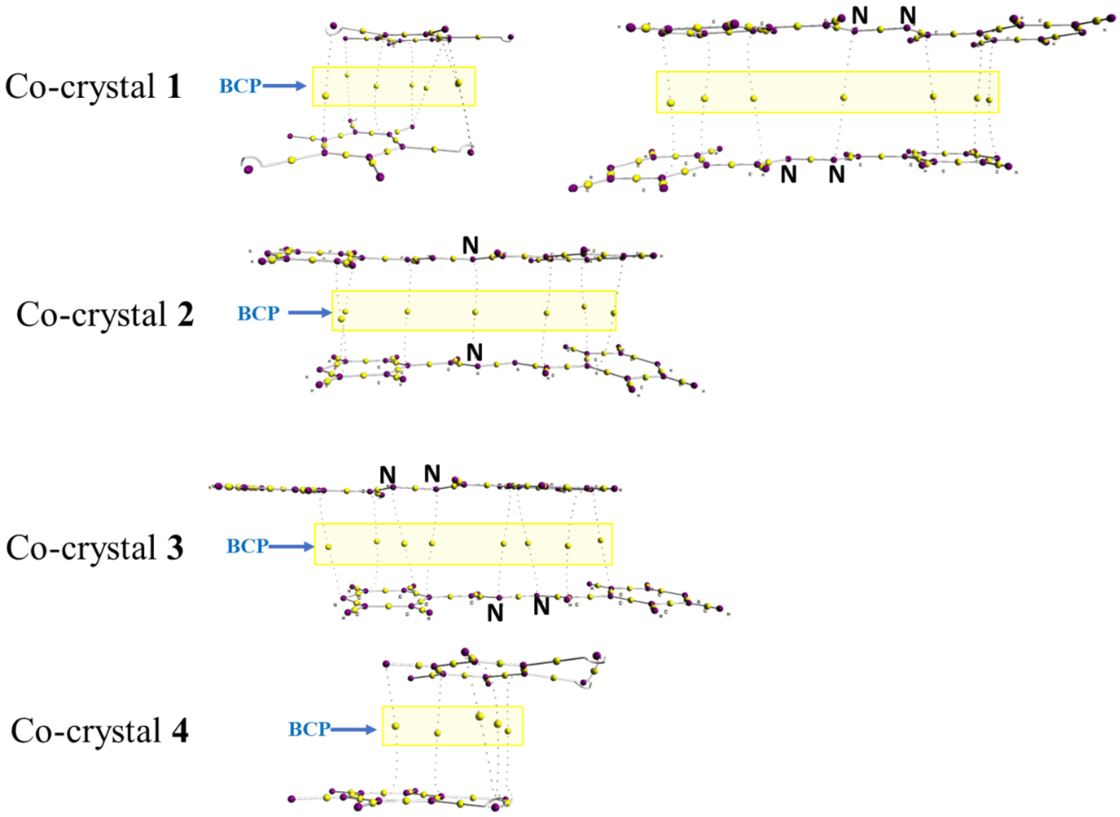

Figure S18: Molecular graphs of stacking in **1,2,3,4** extracted from the corresponding crystals, showing positions of the two or three attractors (purple spheres) linking them bond paths (BPs) and the corresponding bond critical points (BCP) (yellow spheres). The blue arrows indicate the position of the BCP of the intermolecular interactions (IMIs).

Table S1: Geometric parameters of C–X···π interactions in co-crystals **1**–**5**.

Co-crystal	C–X···π	X··· π /Å	(C–X···π)/°
**1**	C17–I2··· π(C13→→C18)	3.8568(19)	70.97(12)
	C33–I6··· π(C31→→C36)	3.8292(19)	70.98(12)
	C14–F3··· π(C13→→C18)	3.537(3)	87.6(2)
	C36–F4··· π(C31→→C36)	3.524(3)	88.0(2)
**2**	C14–F1··· π(C13→→C18)	3.407(4)	141.8(3)
**3**	C15–I2··· π(C13→→C18)	3.617(3)	85.9(2)
	C18–F3··· π(C13→→C18)	3.559(5)	101.2(3)
**4**	C12–I2··· π(C7→→C12)	3.9687(16)	68.68(9)
	C9–F2··· π(C7→→C12)	3.636(3)	84.4(2)
**5**	C4–H4··· π(C7→→C12)	2.95	120
	C12–H12··· π(N1→→C5)	2.91	125

Table S2: π-π stacking interactions of the co-crystal **2**.

**π-π interactions**	**Cg-Cg** (Å)	**α (º)**	**β (º)**	**Slippage** (Å)	**Symmetry operation on Cg**
Cg1-Cg2	3.846(4)	9.5(3)	26.3	1.705	1-X,2-Y,1-Z
Cg2-Cg2	3.853(3)	0.0(3)	19.9	1.314	-X,2-Y,1-Z

Cg1, N1→C1→C2→C3→C4→C5→; Cg2, N4→C10→C9→C8→C12→C11→.

Table S3: Local properties at XBD···XBA interactions (I···N/I···I/I···F) or HBD···HBA (-O-H···N) from QTAIM analysis method, interaction energy (ΔE_{int}) and second order perturbation energies ($E^{(2)}$) from NBO analysis at ωB97XD/def2tzvpp level.

	D···A	$d_{D\cdots A}$ (Å)	ρ_{BCP} $(a.u.)$	∇^2_{BCP} (a.u.)	H_{BCP} $(a.u.)$	ΔE_{int} (kcal/mole)	$E^{(2)}$ (kcal/mol)
1	**I1···N4**	2.89	0.01879	0.0829	0.0012	-5.47	lp_pN1→σ*(C-I4)<br>10.52
	**(I1···N4)-I3···N5**	2.6	0.01694	0.0719	0.00117	-4.98	lp_pN5→σ*(C-I3)<br>8.55
	**I4···N8**	2.9	0.01844	0.0819	0.001120	-6.05	lp_pN8→σ*(C-I4)<br>10.76
	**(I4···N8)-I5-N1**	3.06	0.01431	0.0587	0.00124	-4.74	lp_pN1→σ*(C-I5)<br>6.33
	**I2···F4**	3.029	0.01	0.05209	0.0021635	-1.36	$lp_{sp0.39}$F4→σ*C-I2<br>1.44<br>$lp_{sp0.1}$I2→σ*C-F4<br>0.41<br>lp_pI2→σ*C-F4<br>0.27
	**π···π 4-bpdb**	**6** bond paths with ρ_{BCP} spaning between 0.001-0.0035 a.u.				-9.51	Π(C-C)$_{pyridyl,m1}$→π*(N-C)$_{azine,m2}$<br>0.06<br>Π(C-C)$_{pyridyl,m1}$→π*(C-C)$_{pyridyl,m2}$<br>0.17<br>Π(C-C)$_{pyridyl,m2}$→π*(N-C)$_{azine,m1}$<br>0.08
	**(I2···F4)-I6···F3 trimer**	3.058	0.0092	0.04662	0.0021104	-1.52	$Lp_{sp0.41}$F3→σ*I6-C<br>1,38<br>lp_p F3→σ*I6-C<br>0.19<br>$lp_{sp0.1}$I6→σ*F4-C<br>0.37<br>lp_p I6→σ*F4-C<br>0.17
	**H20···F5**	2.53	0.0051	0.0299	0.001737	-2.39	-
**2**	**N1···I1**	2.83	0.0204	0.0946	0.00122	-6.16	lp_pN1→σ*(C-I1)

							13.9
	**(N1⋯I1)-<br>-I2 ⋯N2<br>trimer**	2.91	0.0181	0.0811	0.00137	-5.63	lp_{p}N2→σ*(C-I2)<br>10.9
	**H10-F3**	2.52	0.0052	0.02906	0.00161	-1.67	lp_{py}F→σ*(C-H)<br>0.06<br>lp_{pz}F→σ*(C-H)<br>0.11
	**π⋯π<br>4-bpdb**		**7** bond paths with ρ_{BCP} spaning between 0.001-0.0035 a.u			-10.14	$\Pi(C\text{-}C)_{pyridyl,m1}$→$\pi^*(N\text{-}C)_{azine,m2}$<br>0.26<br>$\Pi(C\text{-}C)_{pyridyl,m1}$→$\pi^*(N\text{-}C)_{pyridyl,m2}$<br>0.22<br>$\Pi(C\text{-}C)_{pyridyl,m1}$→$\pi^*(C\text{-}C)_{pyridyl,m2}$<br>0.48<br>$\Pi(C\text{-}C)_{pyridyl,m2}$→$\pi^*(N\text{-}C)_{azine,m1}$<br>0.26<br>$\Pi(C\text{-}C)_{pyridyl,m2}$→$\pi^*(N\text{-}C)_{pyridyl,m1}$<br>0.22<br>$\Pi(C\text{-}C)_{pyridyl,m2}$→$\pi^*(C\text{-}C)_{pyridyl,m1}$<br>0.47
	**N1⋯I1**	2.90	0.0188	0.0815	0.001139	-5.56	lp_{p}N1→σ*(C-I1)<br>9.92
	**(N1⋯I1)-<br>-I3⋯N3<br>trimer**	2.99	0.0158	0.0678	0.001339	-5.33	lp_{p}N3→σ*(C-I3)<br>8.34
**3**	**(N1⋯I1)-<br>-(I3⋯N3)<br>I2⋯F3<br>(tetramer)**	2.93	0.01149	0.0609	0.002329	-0.99	$lp_{sp0.42}$F3→σ*(C-I3)<br>2.4<br>$lp_{sp0.1}$I2 → σ*(C-F3)<br>0.63<br>lp_{p}I2→σ*(C-F3)<br>0.2
	**π⋯π<br>4-bpdb**		**8** bond paths with ρ_{BCP} spaning between 0.001-0.0035 a.u			-8.84	$\Pi(C\text{-}C)_{pyridyl,m1}$→$\pi^*(N\text{-}C)_{azine,m2}$<br>0.39<br>$\Pi(C\text{-}C)_{pyridyl,m1}$→$\pi^*(N\text{-}C)_{pyridyl,m2}$<br>0.06<br>$\Pi(C\text{-}C)_{pyridyl,m2}$→$\pi^*(N\text{-}C)_{azine,m1}$

							0.38<br>$\Pi^*(C\text{-}N)_{p,m2} \rightarrow \pi^*(C\text{-}C)_{p,m1}$<br>1.16
**4**	**N1⋯I1**	2.86	0.0194	0.0876	0.00121	-6.14	$lp_p N1 \rightarrow \sigma^*(C\text{-}I1)$<br>12.34
	**I1⋯I2**	3.88	0.0065	0.0022	0.00096	-3.3	$Lp_{pz} I1 \rightarrow \sigma^*(C\text{-}I2)$<br>3.40
**5**	**O-H⋯N**	1.93	0.03144	0.13264	0.00106	-6.99	$lp_{sp2.23} N \rightarrow \sigma^*$ (O-H)<br>10.84
